# Identification of New Potential Inhibitors of Quorum Sensing through a Specialized Multi-Level Computational Approach

**DOI:** 10.3390/molecules26092600

**Published:** 2021-04-29

**Authors:** Fábio G. Martins, André Melo, Sérgio F. Sousa

**Affiliations:** 1 UCIBIO/REQUIMTE, BioSIM—Departamento de Biomedicina, Faculdade de Medicina da Universidade do Porto, 4200-319 Porto, Portugal; fabmaru@gmail.com; 2 LAQV/REQUIMTE, Departamento de Química e Bioquímica, Faculdade de Ciências da Universidade do Porto, 4169-007 Porto, Portugal; asmelo@fc.up.pt

**Keywords:** biofilms, *Chromobacterium violaceum*, CviR, quorum-sensing, virtual screening, molecular docking, molecular dynamics simulations, MM/PBSA, MM/GBSA

## Abstract

Biofilms are aggregates of microorganisms anchored to a surface and embedded in a self-produced matrix of extracellular polymeric substances and have been associated with 80% of all bacterial infections in humans. Because bacteria in biofilms are less amenable to antibiotic treatment, biofilms have been associated with developing antibiotic resistance, a problem that urges developing new therapeutic options and approaches. Interfering with quorum-sensing (QS), an important process of cell-to-cell communication by bacteria in biofilms is a promising strategy to inhibit biofilm formation and development. Here we describe and apply an in silico computational protocol for identifying novel potential inhibitors of quorum-sensing, using CviR—the quorum-sensing receptor from *Chromobacterium violaceum*—as a model target. This in silico approach combines protein-ligand docking (with 7 different docking programs/scoring functions), receptor-based virtual screening, molecular dynamic simulations, and free energy calculations. Particular emphasis was dedicated to optimizing the discrimination ability between active/inactive molecules in virtual screening tests using a target-specific training set. Overall, the optimized protocol was used to evaluate 66,461 molecules, including those on the ZINC/FDA-Approved database and to the Mu.Ta.Lig Virtual Chemotheca. Multiple promising compounds were identified, yielding good prospects for future experimental validation and for drug repurposing towards QS inhibition.

## 1. Introduction

Bacterial biofilms are aggregates of microorganisms anchored to a surface and embedded in a self-produced matrix of extracellular polymeric substances [1]. Due to the properties of the matrix and to the intercellular interactions between the bacteria within, the biofilm develops, thus becoming increasingly sophisticated and offering the constituting bacteria several advantages over their planktonic counterparts [2,3]. These include an increased antibiotic resistance, elevated levels of lateral gene transfer, higher stress resistance and subversion of host defense mechanisms [4,5,6]. Quorum sensing (QS), a process of cell-to-cell communication, is important for the control of several virulence factors and plays a key role during biofilm formation. In this process, cells communicate using auto-inducer signals. QS allows bacteria to synchronously adjust gene expression to alter their behavior in response to changes in population density and the surrounding bacterial community [7].

Several clinically important pathogenic bacteria, such as cystic fibrosis-associated *Pseudomonas aeruginosa*, surgical sites associated with *Staphylococcus aureus* and many others, cause infection through biofilm formation [8]. This can have devastating consequences. Microbes that reside in biofilms may not be eliminated by traditional antibiotics because of metabolic dormancy or molecular resistance mechanisms [9]. The US National Institutes of Health estimate that 80% of all bacterial infections occurring in the human body are biofilm-related [10]. Therefore, the overall burden of biofilm infections is significant, and it has been recognized as a serious threat to our society [10]. Biofilm infections are not easily treated with existing antimicrobial approaches because the biofilm recalcitrance is a consequence of its complex physical and biological properties [11]. QS signaling plays an important role in biofilm formation in such a way that specific QS signaling blockage is an effective way to prevent the biofilm formation of most pathogens. Additionally, QS inhibition does not affect the normal growth of the bacteria. Therefore, they do not create any evolutionary pressure for the emergence of multidrug-resistant bacteria. Consequently, QS inhibitors usually have a longer functional shelf life than modern antibiotics and are regarded as a promising therapeutic alternative in combined therapies [12,13].

*Chromobacterium violaceum* is a large, motile, Gram-negative bacillus, which lives on soil and water in tropical and subtropical regions, and it can act as an opportunistic pathogen for animals and humans. It enters through broken skin by contamination with soil or stagnant water [14]. There have been reports of it causing localized skin and soft tissue infection and systemic or invasive infection. These include necrotizing fasciitis, visceral abscesses, osteomyelitis, and central nervous system disease [15]. Infections due to *C. violaceum,* albeit relatively rare, with less than 150 published clinical reports, are associated with high mortality [16].

This bacterium is known for the production of a natural violet pigment with antibiotic properties, known as violacein, whose production is regulated via quorum-sensing [17]. Since this QS-regulated trait is an easily observable and quantifiable trait, *C. violaceum* is widely used as a model organism for QS research [18]. The QS system in *C. violaceum* is homologous of the LuxI/LuxR system found *in Vibrio fischeri*, with the AHL synthase being CviI and the transcriptional activator being CviR [19,20].

**Figure 1:**
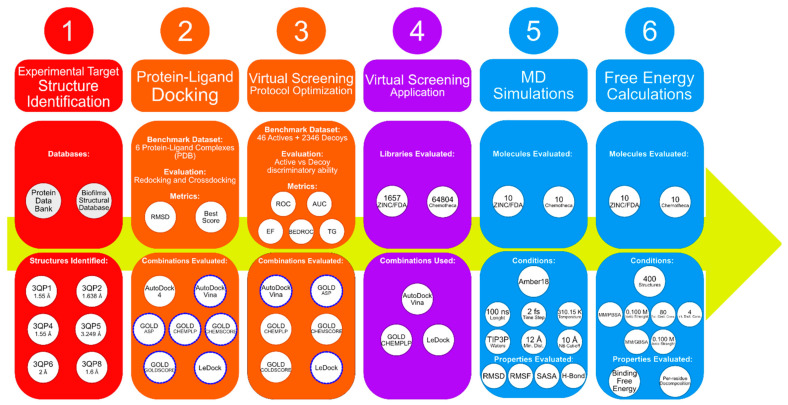
Scheme depicting the workflow of this work.

Over the years, the usage of computer-aided drug design (CADD) as a preliminary stage of drug design has increased, finding application in the study of many problems, including in the search for quorum-sensing inhibitors [21,22,23,24,25,26,27,28,29,30,31,32,33,34,35,36]. This makes the entire process more cost-efficient and minimizes failures [37]. Therefore, the aim of this study is to use CADD to understand the binding determinants and particularities of the CviR-binding pocket as a QS inhibition model target and to identify new promising molecules for blocking quorum-sensing and preventing biofilm formation. Different computational methods were combined in this study, including protein–ligand docking, virtual screening, MD simulations and MM/PB(GB)SA calculations. Figure 1 depicts the workflow of this work.

## 2. Materials and Methods

### 2.1. PDB Structures Identification

The structures were identified using the Biofilms Structural Database [38] and then downloaded from the Protein Data Bank [39]. These X-ray structures and the corresponding resolution value, ligand and bacterial strain are summarized in Table 1.

The CviR structures belong to two different strains. Additionally, different ligands with different activities are complexed with each structure. 3QP1 is complexed with its native ligand C6-HSL, which is a full agonist in strain 31532. 3QP2 and 3QP4 are bonded to ligands with longer acyl chains, which fail to fully activate CviR. C8-HSL leads to 40% of the original activity, and C10-HSL elicits only 6%. These ligands also work as a partial antagonist in the presence of C6-HSL. 3QP5 is bonded to chlorolacetone, an even stronger antagonist than C10-HSL. 3QP6 is bonded to C6-HSL, which functions as an antagonist on strain 12472 [20]. Finally, 3QP8 is bonded to C10-HSL. This is an agonist, which is located closer to this strain’s native ligand (3-hydroxy-C10-HSL). The proteins from each strain are 87% identical in the amino acid sequence, with its difference in autoinducer being partially due to a naturally occurring serine instead of a methionine at position 89. This is a key residue that occupies the opening of the ligand-binding pocket. The importance of Met 89 is reinforced by the fact that, when using antagonists, the side-chain swings away. The extension of this movement increases with the strength of the antagonist. It was observed that Met89′s side-chain swings away from the ligand-binding pocket along with an increase in length of the ligand’s acyl chain. The extent of this variation relates to the antagonist capabilities of the ligand. 3QP1 is bonded to the native ligand, C6-HSL, and Met89 is in its original pose. In 3QP2, there are two conformations: one similar to 3QP1 and the other in which there is a small, intermediate variation on the side-chain orientation. In both 3QP4 and 3QP5, the side-chain has fully changed its position, swinging far away from the center of the binding pocket [20]. The receptors were then isolated from the ligands using PyMOL [40]. In the case of 3QP5 and 3QP8, it was also necessary to isolate one of the four chains that are in the PDB file. In both cases, chain A was the one selected.

### 2.2. Protein-Ligand Docking Protocol Validation

For the validation of the molecular docking protocol, redocking and cross-docking studies are performed. The goal of the redocking studies is to see if the molecular docking protocol can accurately reproduce the experimental complexed pose of the known ligands. Several parameters, such as the size and coordinates of the search area, are evaluated, and the results were compared. The RMSD between the re-docked and the original poses was calculated to better evaluate the results. In cross-docking studies, each ligand from each structure is docked into all the other structures. Considering that all structures are of the same protein, this is an assessment of how accurately each structure can accommodate in its pocket the ligands from the other structures, i.e., its general usefulness [41]. As the performance of different docking programs/scoring functions is known to depend among other features on the specific type of target under consideration [42], in this study, four popular protein–ligand docking programs [43,44] and seven different scoring functions were taken into consideration: AutoDock 4 [45]; AutoDock Vina (Vina) [46]; LeDock [47]; and GOLD (with the scoring functions CHEMPLP, ASP, CHEMSCORE and GOLDSCORE) [48]. The RMSD calculations were done using DockRMSD [49]. For AutoDock and Vina, the search area was a box designed using the AutoDock/Vina plugin for PyMol by Daniel Seeliger [50]. This search area was based on the residues known to be involved in the interaction between the ligand and CviR. These are Tyr80, Trp84, Asp97 and Ser155. For LeDock, the same box dimensions were considered. With the different GOLD scoring functions, the search area considered involved spheres with the same center coordinates and volume as in AutoDock, Vina and LeDock. With each docking program/scoring function, the protocol was optimized for each protein target to minimize the RMSD in the redocking predictions of the reference ligand by comparison with the crystallographic structure of the corresponding complex. The optimized parameters for each program/scoring function were: AutoDock– docking box position, docking box dimensions; Vina—docking box position, docking box dimension, exhaustiveness; LeDock—docking box position, docking box dimension; GOLD (CHEMPLP, ASP, CHEMSCORE, and GOLDSCORE)—binding pocket center, docking region radius, search efficiency, number of runs.

It should be noted that the different docking program results have different values and scales. The score for all the GOLD scoring functions is dimensionless, and the higher the score, the better the binding affinity. AutoDock, Vina and LeDock scoring functions, on the other hand, use a metric that resembles binding free energy (in kcal/mol), so a more negative value means a better-predicted affinity.

The best combinations of protein-ligand/scoring functions were used in the next stage.

### 2.3. Virtual Screening Protocol Optimization

The optimization of the virtual screening protocol was performed via a virtual screening benchmark test on the CviR system, considering a library of experimentally confirmed active molecules for CviR and randomly generated decoys [42], i.e., molecules with similar 1-D physicochemical properties to known actives (e.g., molecular weight, calculated LogP), but dissimilar 2-D topology to be likely non-binders.

A total of 46 actives (Figure S1 in the supplementary materials) were used, 23 obtained from ChEMBL [51] and 23 found in the literature [28,52,53,54,55,56,57,58,59,60,61,62]. Structures were downloaded from PubChem [63] or modeled using the Avogadro molecular editing program [64]. These are listed in the supplementary materials. To obtain the decoys, the 46 actives were submitted to the Database of Useful Decoys– Enhanced (DUD-E) server [65]. DUD-E generated 50 decoys per each active. This led to a final library of 2346 molecules, constituting a CviR-specifically designed and challenging test set to evaluate and optimize the performance of different virtual screening protocols in their ability to discriminate between CviR binders and non-binders.

Different metrics are routinely used to evaluate the performance of VS protocols, including receiver operating characteristics (ROC) curves and the respective area under the curve (AUC), enrichment factor (EF), robust initial enhancement (RIE), Boltzmann enhanced discrimination of ROC (BEDROC), predictive curves (PC) and total gain (TG) [42,66]. The calculation of the metrics to evaluate the performance of VS protocols was performed using two different applications. The ROC and enrichment factors were calculated on Microsoft Excel. The web-based application Screening Explorer [66] was used for calculating Total Gain and BEDROC.

In this step, Vina, all scoring functions of GOLD and LeDock were used, with Vina, GOLD with the CHEMPLP scoring function (GOLD/CHEMPLP) and LeDock being the best performing VSs. The optimized parameters for Vina were an exhaustiveness parameter of 8 and a search box with the following dimensions: 15.0 × 15.0 × 14.0 Å. For GOLD, the binding pocket radius was set to 9.1 Å, and the number of independent genetic algorithms (GA) runs set to 10. For each GA run, the population size was 100 with a selection pressure of 1.1. The number of operations was 100,000, with the crossover, mutation and migration frequency being 95, 95 and 10, respectively. For LeDock, a clustering by RMSD was performed, with a value of 1 Å set as the threshold, and the number of selected binding poses was 20.

### 2.4. Virtual Screening for the Identification of New Potential CviR Binders

Three optimized virtual screening protocols selected in the previous stage and based on Vina, GOLD/CHEMPLP and LeDock were used for identifying new potential CviR binders. Two virtual databases were considered: the US Food and Drug Administration (USFDA) approved database available on ZINC [67], and the Mu.Ta.Lig. Virtual Chemotheca [68]. ZINC is a public access database, which is used to obtain compounds for several uses. These include virtual screening, ligand discovery and force field development. It was created in the Department of Pharmaceutical Chemistry at the University of California (UCSF) and included 220 million molecules [67]. In the present work, 1657 FDA-approved molecules were available in this database and were assessed in this work to evaluate the possibility of drug repurposing. The Mu.Ta.Lig. (Multi-Target Ligand) Chemotheca database was created from COST Action CA15135—Multi-target paradigm for innovative ligand identification in the drug discovery process and was developed with the goal of identifying multi-target agents and repurposing known compounds. A large number of molecules with promising pharmaceutical relevance are developed every year and are forgotten when they fail to have the desired effect. However, these molecules can have a positive effect on other targets. At the time of this work, Mu.Ta.Lig Virtual Chemotheca features 64,804 compounds [68].

From the virtual screening results, a selection of 10 molecules from the FDA-approved database and 10 molecules from the Mu.Ta.Lig Virtual Chemotheca was done among the top results in all VSs, taking into consideration the chemical diversity and predicted physical-chemical properties of the molecules.

### 2.5. Molecular Dynamics (MD) Simulations

Molecular dynamics (MD) simulations were carried out using the Amber18 software package [69]. Molecular mechanics parameters for the selected molecules were assigned using ANTECHAMBER and considering the general amber force field [70], with RESP charges calculated using HF/6-31G(d) with Gaussian16 [71]. The protein was described through the amber14sb force field [72]. The protein–ligand complexes were placed into a box of TIP3P water molecules, whose edges are placed at least 12 Å away from each atom of the complex. All the MD were performed using periodic boundary conditions. Long-range electrostatic interactions were calculated using the particle mesh Ewald summation method. The cutoff value for the short-range electrostatic and Lennard–Jones interactions was set at 10.0 Å. All bonds involving hydrogen atoms were constrained using the SHAKE algorithm, enabling applying a 2 fs time step.

All ligand-CviR solvated complexes went through 4 minimization steps, with a maximum of 2500 cycles each. After 1250 cycles, the minimization method was switched from steepest descent to conjugate gradient. In the first minimization, all molecules except water molecules were restrained. In the second minimization, only hydrogen atoms were not restrained. During the third minimization, only the protein backbone was restrained. Finally, for the last minimization step, there were no restraints. Following the minimization phase, all solvated complexes were heated from 0 to 310.15 K over 50 ps. They were further equilibrated at 310.15 K during 50 ps to stabilize the density. Finally, for each ligand-CviR complex, the production phase was run for a total of 100 ns in an NPT ensemble at a pressure of 1 bar and a temperature of 310.15 K.

The analysis of the trajectories was carried out using the cpptraj tool [73] and VMD [74].

### 2.6. MM/PB(GB)SA Calculations and Replica MDs

The MM/PB(GB)SA calculations were performed using the MM/PBSA.py script available in AMBER [75]. The calculations considered the last 40 ns of the MD simulation of every complex, using an interval of 100 ps. This means that the program used every 10th frame of the simulation. In the MM/PBSA calculations, the following constants were used: ionic strength of 0.100 mol dm^−3^, the external dielectric constant of 80.0 and the internal dielectric constant of 4. In the MM/GBSA calculations, a salt concentration of 0.100 mol dm^−3^ was used.

The Free energy decomposition option was used to obtain information about the local interactions of the complex. Using per-residue decomposition, the contribution of each residue to the total free energy was estimated.

For the 4 ligands with the strongest binding free energy identified, 3 additional 100 ns replica molecular dynamics simulations were performed, using the protocol and approximation described in 2.5.

## 3. Results and Discussion

Table 2 compares the best redocking performance obtained with Autodock4, Vina, CHEMPLP, CHEMSCORE, GOLDSCORE, ASP and LeDock in terms of RMSD between the predicted pose and the X-ray crystallographic pose. One important detail is that 3QP2 was separated into two different files, one for each conformation of the important residue Met89. 3QP2a has the intermediate conformation, while 3QP2b has a similar conformation to 3QP1.

The RMSD values show that the best docking program for reproducing experimental poses of the ligand is LeDock, with an average RMSD of 0.74 Å. All scoring functions from GOLD also have a good performance. On the other hand, the programs, which performed worse were Vina and AutoDock 4, with an average RMSD of 1.44 Å and 1.13 Å, respectively. Vina has the worse overall RMSD value for the redocking of 3QP6. In this case, the program placed the molecule the opposite way when compared to the crystallographic structure. However, this could be due to the fact that a smaller molecule is being docked in a binding pocket previously accommodating a larger ligand. In reality, when C6-HSL binds to this structure (in this strain, C6-HSL functions as a partial antagonist), there is a shift in the DNA-binding domain, which will help fill the binding pocket [20]. Nevertheless, most RMSD values are below 2 Å, which indicates a good overall performance from all the different molecular docking software/scoring functions. This good performance is mainly seen with the lactone head group, with most programs struggling with the acyl chain. The scores from all redocking procedures are available in Table S1 in the supplementary materials.

The cross-docking studies (details on Tables S2 and S3 in the supplementary materials) show that, on average, the best results are seen on structures 3QP6 and 3QP8, both from strain 12472. From strain 31532, the structure, which generates the best scores, is 3QP4. In contrast, the worst results in all ligands and software are seen on structure 3QP5. This may be due to the worse resolution of this structure. Similar to the redocking studies, C6-HSL shows less affinity when compared to all other ligands, while C10-HSL and CL show the best results. This behavior is shown in most structures and software. One major difference from the redocking and cross-docking studies is the behavior of 3QP6. As expected, the bad performance during the redocking studies was due to the ligand, C6-HSL. When docking other ligands, 3QP6 is frequently the best structure that leads to the best results.

Globally, the redocking and cross-docking studies suggest that most combinations of docking program/scoring function, when optimized, perform similarly. For these structures, the poorest results were obtained with AutoDock 4 that is also the most time-consuming program.

Regarding the structures, the main conclusions emerging from these results are that both structures from strain 12472 (3QP6 and 3QP8) displayed the highest scores with all scoring functions, from which 3QP6 is the structure that generated the best results. From strain 31532, 3QP4 displayed the higher and most consistent scores for all scoring functions, while 3QP1, 3QP2a and 3QP2b presented more variable scores. Finally, 3QP5 was consistently the worst performing of all available structures of CviR.

After evaluating and optimizing the docking of known CviR ligands with well-characterized and experimentally defined binding poses, the next step was to evaluate the ability of this protocol in distinguishing between known CviR binders and non-binders.

Table 3 and Table 4 compare the ability of Vina, CHEMPLP, CHEMSCORE, GOLDSCORE, ASP, LeDock, as well as all the different PDB structures, in distinguishing experimentally confirmed CviR binders (actives) and non-binders (decoys) through the usage of several different evaluation metrics.

The best-performing programs were consistent across most of the metrics, with Vina and GOLD with the CHEMPLP scoring function (GOLD/CHEMPLP) being more capable of distinguishing the experimentally confirmed binders from the decoys, generally followed by LeDock. With this in mind, the programs used in the virtual screening stage of this work were Vina, GOLD/CHEMPLP and LeDock (see Figure 1).

Finally, it is important to select the receptor structures. Across nearly all metrics, the best discrimination ability between binders and non-binders was obtained when using 3QP6 and 3QP8. However, since they are both from strain 12472, the decision was made to use the best performing structure from each strain. The structure from strain 31532 that generally generated the best results was 3QP4. Therefore, the two CviR structures used in the virtual screening step of this work were 3QP4 and 3QP6.

**Figure 2:**
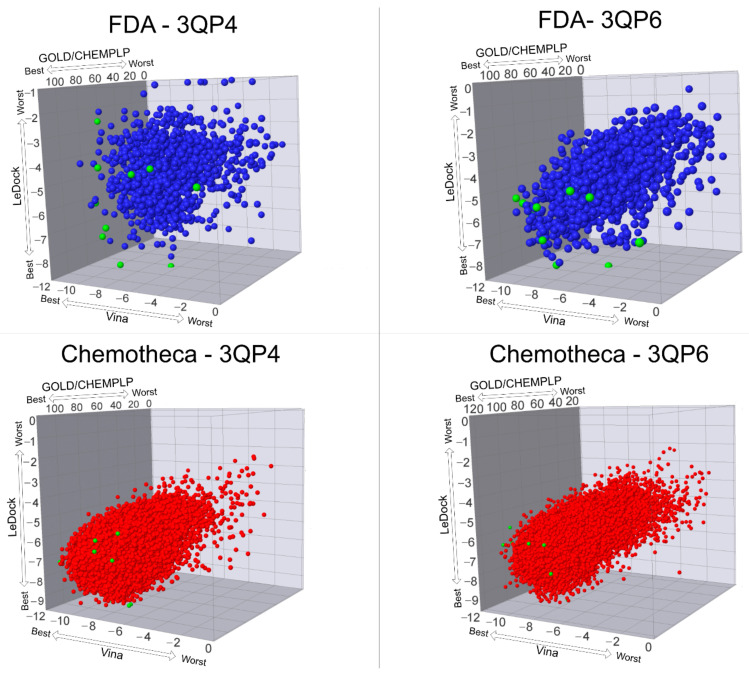
Results of all virtual screening procedures. The green-colored molecules are the ones selected for the molecular dynamics simulations.

The optimized protein–ligand docking and virtual screening protocols were then applied to two libraries of compounds: the US Food and Drug Administration (USFDA) approved database available on ZINC (ZINC/FDA) to consider possible drug repurposing alternatives, and the more general Mu.Ta.Lig. Virtual Chemotheca. The results of all virtual screenings are in Figure 2.

**Figure 3:**
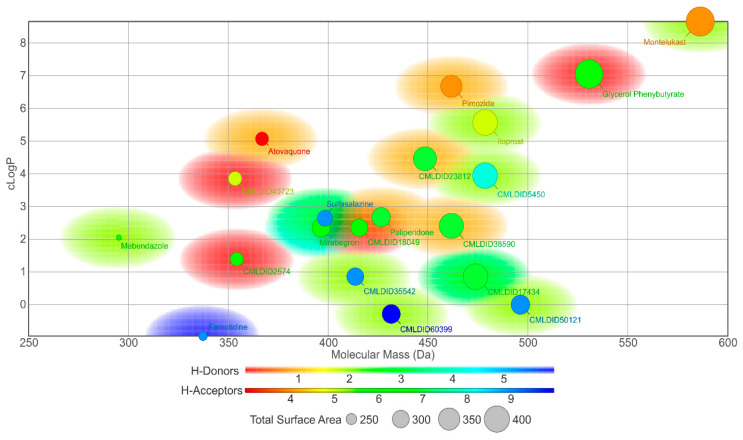
Graph displaying the variety of the 20 molecules selected from the virtual screening procedures by displaying Table 4. 2D structures of the best performing compounds from the virtual screening of the ZINC/FDA library and Mu.Ta.Lig Virtual Chemotheca.

In this study, different molecular docking programs and different PDB structures were used in a consensus approach. This approach can explore many potential ligands with greater accuracy, contributing to a more rigorous selection of the molecules for testing. From the top solutions in the ranked lists obtained from the VS, 10 molecules from each library were selected for the MD simulations. For each library, the selection was based on the position of the molecules on the different VS protocols. The choice took into consideration the following criteria: (i) molecules among the top 25 solutions for the two structures considered in the VS; (ii) molecules among the top 25 solutions with more than one scoring function; (iii) molecular diversity taking into consideration the physical-chemical properties (Figure 3).

**Figure 4:**
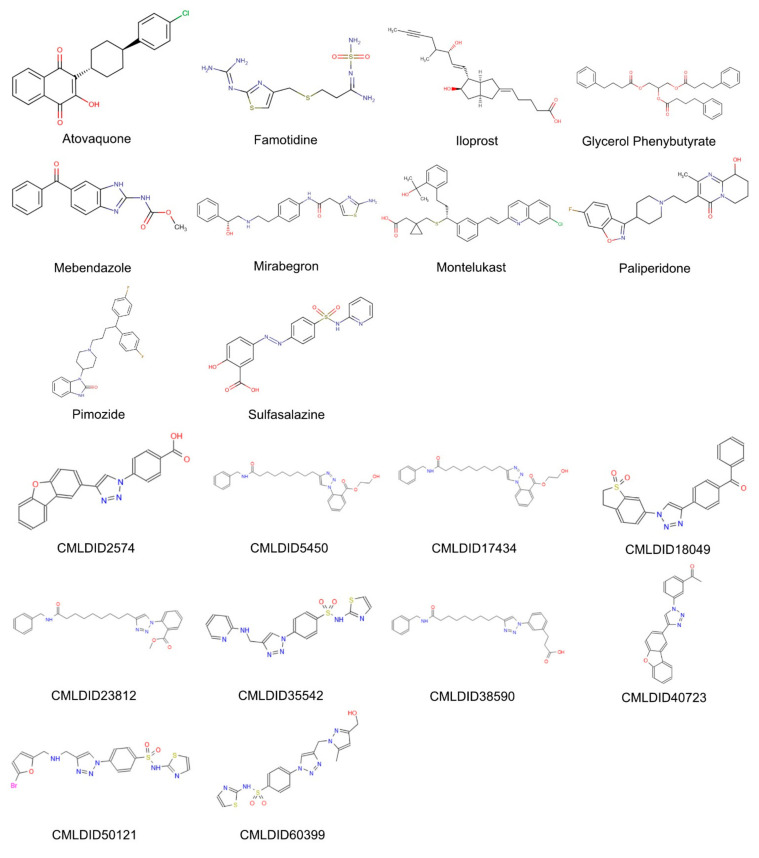
2D structures of the best performing compounds from the virtual screening of the ZINC/FDA library and Mu.Ta.Lig Virtual Chemotheca.

The molecules from the ZINC/FDA database were atovaquone, famotidine, iloprost, mebendazole, mirabegron, montelukast, paliperidone, pimozide, glycerol phenylbutyrate, and sulfasalazine (Figure 4). The molecules from Chemotheca were CMLDID2574, CMLDID5450, CMLDID17434, CMLDID18049, CMLDID23812, CMLDID35542, CMLDID38590, CMLDID40723, CMLDID50121 and CMLDID60399 (Figure 4). As a display of the variability of the selected molecules, multiple important properties (cLogP, molecular mass, total surface area, hydrogen donors and acceptors) of the 20 selected molecules are displayed in Figure 3. These properties and graphs were calculated using the data visualization and analysis program, DataWarrior [76]. Comparisons between the score of the 20 selected molecules and the scores obtained by the known active molecules is available in Figures S2–S7 in the supplementary materials.

Based on the EF 1% for the actives/decoys training set, we estimated the minimum number of actives expected to be found on the 25 first positions of each VS (Table 5).

The next step of this work involved 100 ns of molecular dynamics simulation for each of the above-mentioned molecules in complex with 3QP6. This was done to validate the protein–ligand docking results, to evaluate the structural stability of the protein–ligand complex and to carry out the MM/PB(GB)SA calculation. For reference, MD simulations of 3-hydroxy-C10-HSL and C10-HSL in complex with the protein were also performed. While the native ligand of the strain 12472 of *C. violaceum* is 3-hydroxy-C10-HSL, C10-HSL is an agonist for this protein [20,77]. Additionally, this ligand was the agonist that generated the highest scores on the cross-docking studies with 3QP6. Consequently, C10-HSL, together with 3-hydroxy-C10-HSL, was selected as the reference ligands. Since these MD simulations were used for the refinement of the virtual screening results, only the ligand-binding domain was considered. In the future, we intend to perform further studies for a better understanding of how these ligands could affect the DNA-binding domain.

To assess the structural stability of the complexes, RMSD calculations were performed for the Cα atoms of each complex and for the ligands.

Considering the selected ligands from the ZINC/FDA database and from the Chemotheca database, all complexes exhibit low RMSD values through the simulation. Most ligands also display low RMSD values than the docking prediction (Table 6). However, multiple molecules display higher values, suggesting an induced-fit adjustment to the CviR-binding pocket during the MD simulation. The low standard deviation confirms that after the initial 40 ns of simulation, the ligand positions are well stabilized. This is further confirmed by the solvent-accessible surface area analysis. An increase in SASA from the initial pose predicted from docking would imply that the ligand was exiting the binding pocket. Fortunately, all ligands display a stable SASA value along the simulation. This, together with the RMSD results, confirms that all the selected ligands form stable complexes with CviR. Further information on the MD simulations is available in the supplementary materials (Figures S8–S11 and Table S4).

**Figure 5:**
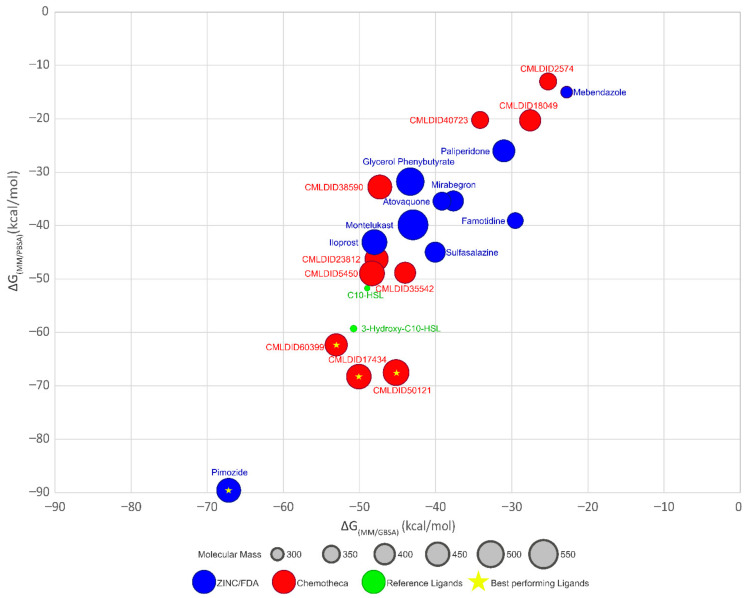
Graph displaying and comparing the results of the free-energy calculation using MM/PBSA and MM/GBSA methods. Blue ligands are the ligands obtained from the ZINC/FDA database, while red ligands are part of Chemotheca. The reference ligands are in green. The best performing ligands are highlighted with a star.

The final step of this study was to perform a MM/PB(GB)SA analysis for each ligand–receptor complex to determine the binding free energy of each ligand. For these calculations, only the last 40 ns of MD simulation were considered. Results are presented in Table 7 and Figure 5.

The results demonstrate that most ligands present a lower affinity towards CviR than the reference ligands (MM/GBSA predicted values between −22.7 ± 0.1 and −67.2 ± 0.2, against −49.0 ± 0.2 and −51.0 ± 0.2 kcal/mol and MM/PBSA predicted values between −13.0 ± 0.1 and −89.6 ± 0.2, against −51.7 ± 0.2 and 59.6 ± 0.2 kcal/mol) The only ligand from the ZINC/FDA database, which displays higher affinity towards CviR is pimozide (−67.2 ± 0.2 and −89.7 ± 0.2 kcal/mol, respectively). Pimozide is used as an antipsychotic agent and for the suppression of vocal and motor tics in patients with Tourette syndrome. Although its exact mechanism of action is unknown, it is thought that it inhibits the dopamine D2 receptor [78].

For the molecules from Chemotheca, only CMLDID17434 (−68.1 ± 0.2 kcal/mol for MM/PBSA) and CMLDID60399 (−53.0 ± 0.2 kcal/mol for MM/GBSA and −61.8 ± 0.2 kcal/mol for MM/PBSA) with both methods, and CMLDID50121 using MM/PBSA (−67.4 ± 0.2 kcal/mol) display higher affinity towards CviR than C10-HSL and 3-hydroxy-C10-HSL.

To provide additional confirmation of these results, and to better assess if the best performing ligands are indeed true binders, replicate MD runs were performed. For each of the four best-performing ligands, three additional MD simulations were performed, and the average RMSD, RMSF, SASA and number of H bonds were calculated. These results are shown in Table 8.

Across all evaluated metrics, the replicas display similar results to the original runs. Furthermore, the percentage of SASA buried on all replicas gives additional strength to the prediction that these ligands are true binders and remain tightly bound.

**Figure 6:**
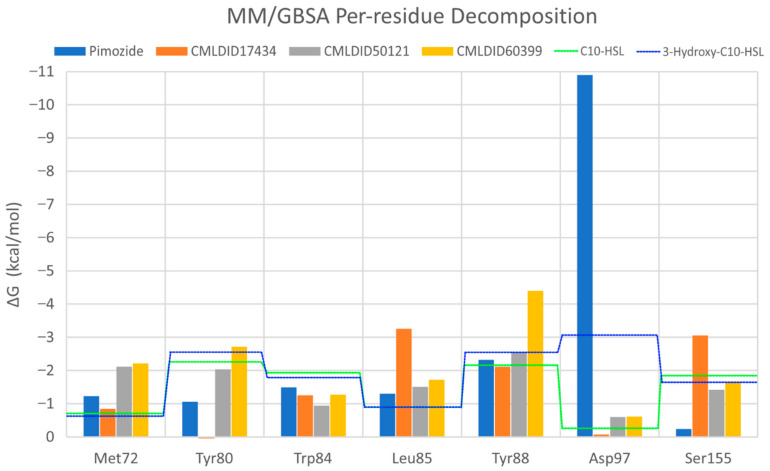
Per-residue decomposition of the free energy calculations using MM/GBSA for the best performing ligands. Table 3. hydroxy-C10-HSL; the green dotted line represents C10-HSL.

**Figure 7:**
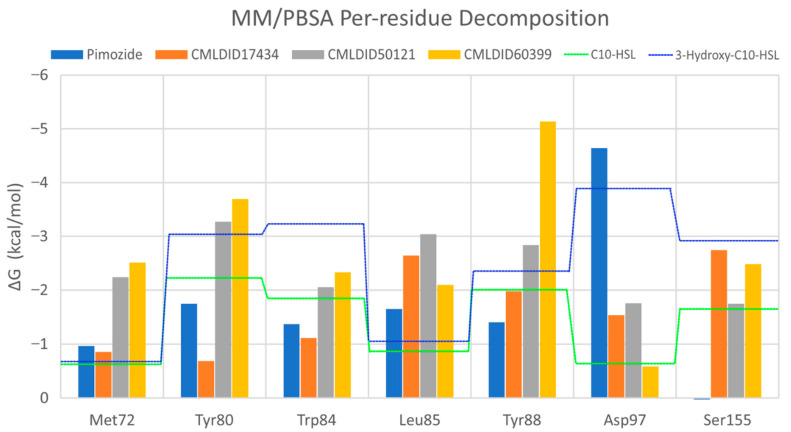
Per-residue decomposition of the free energy calculations using MM/PBSA for the best performing ligands. The blue dotted line represents 3-hydroxy-C10-HSL; the green dotted line represents C10-HSL.

**Figure 8:**
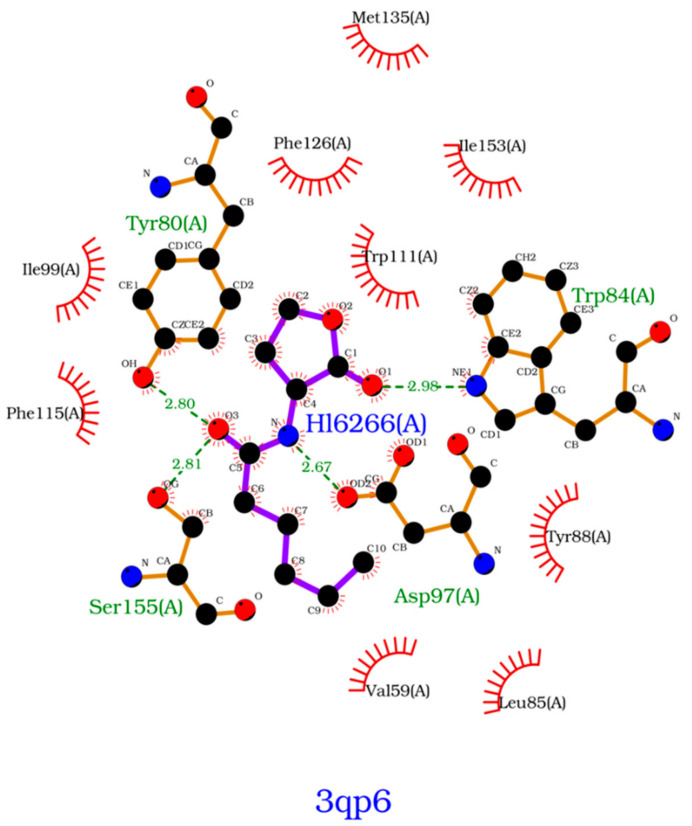
Interactions map, obtained with LigPlot+, between C6-HSL and 3QP6.

To further analyze the affinity between the ligands and the receptor, the overall Gibbs energy of association was decomposed into the contribution associated with each residue (Figure 6 and Figure 7). On 3-hydroxy-C10-HSL, the residues with the higher contribution to the Gibbs energy of association are Tyr80, Trp84, Tyr88, Asp97 and Ser155. As for C10-HSL, using both methods, the amino acids that have a bigger impact are Tyr80, Trp84, Tyr88 and Ser155. When comparing with the already known interactions map obtained with LigPlot+ [79] (Figure 8), these results show great similarity. The only difference is the contribution of Asp97 in the interaction with C10-HSL. While this residue is considered to have an important role in the binding of ligand to CviR, the calculations show a less favorable result for Asp97.

Overall, the residues with the greatest contribution for the affinity of the ligands towards CviR are Met72, Tyr80, Trp84, Leu85, Tyr88 and Ser155. For pimozide, the bigger contribution is obtained from Tyr80 (MM/PBSA), Tyr88 (MM/GBSA), and Asp97. Whereas on most ligands, the calculations show a less favorable contribution from this residue, with pimozide, it is the major contribution for the affinity between the ligand and the protein. For CMLDID17434, the residues with the highest contribution to the overall result using both methods are Leu85, Tyr88 and Ser155. In the case of CMLDID50121, Tyr80, Leu85 and Tyr88 are the residues with the most impact in the predicted affinity towards CviR. Lastly, for CMLDID60399, the amino acids with the greater impact are Met72, Tyr80, Tyr88.

**Figure 9:**
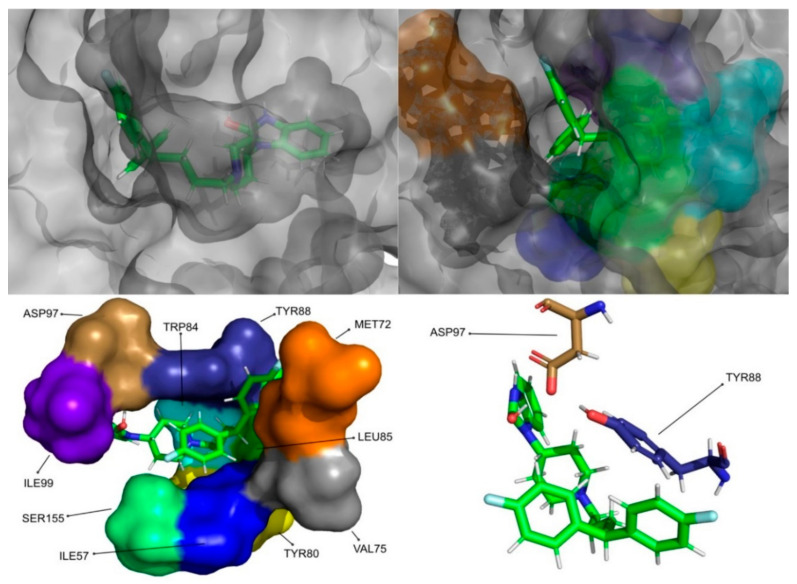
Pimozide in complex with CviR. The top left picture represents the ligand in licorice and the protein on the surface. The top right and bottom left pictures feature, on the surface, the amino acid residues, which, overall, have a bigger impact on the predicted affinity. The bottom right picture depicts the ligand and the amino acids with the biggest contribution to the affinity represented in licorice.

**Figure 10:**
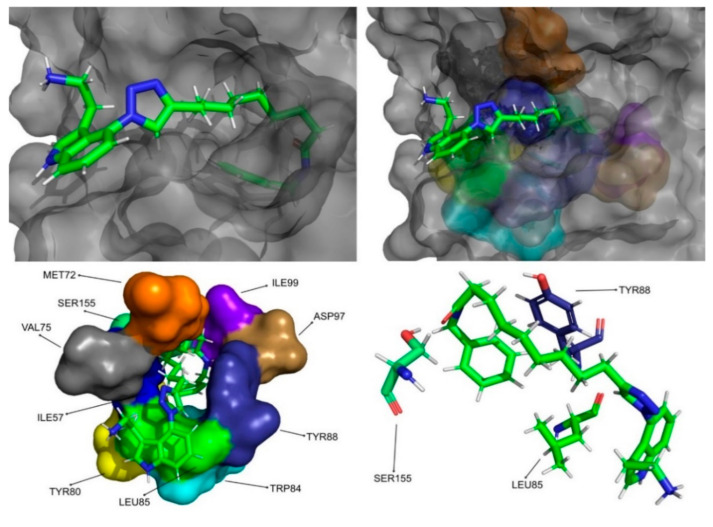
CMLDID17434 in complex with CviR. The top left picture represents the ligand in licorice and the protein on the surface. The top right and bottom left pictures feature, on the surface, the amino acid residues, which, overall, have a bigger impact on the predicted affinity. The bottom right picture represents the ligand in licorice. The bottom right picture depicts the ligand and the amino acids with the biggest contribution to the affinity represented in licorice.

**Figure 11:**
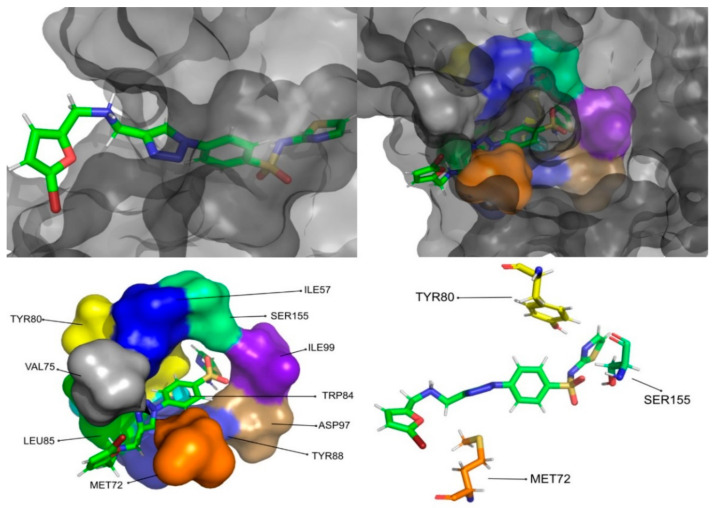
CMLDID50121 in complex with CviR. The top left picture represents the ligand in licorice and the protein on the surface. The top right and bottom left pictures feature, on the surface, the amino acid residues, which, overall, have a bigger impact on the predicted affinity. The bottom right picture represents the ligand in licorice. The bottom right picture depicts the ligand and the amino acids with the biggest contribution to the affinity represented in licorice.

**Figure 12:**
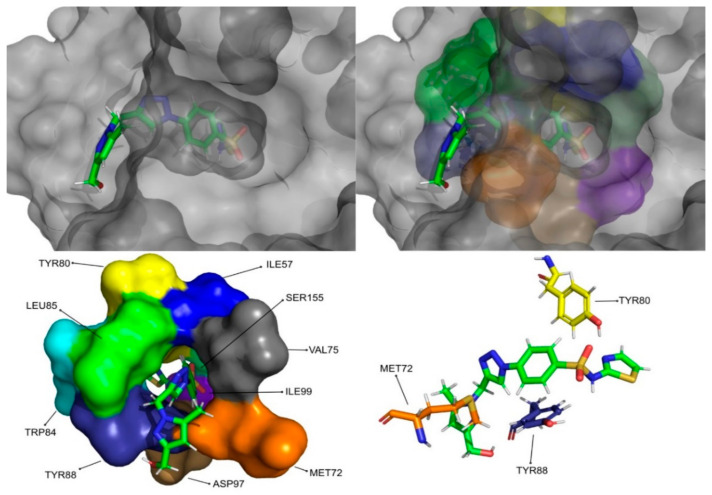
CMLDID60399 in complex with CviR. The top left picture represents the ligand in licorice and the protein on the surface. The top right and bottom left pictures feature, on the surface, the amino acid residues, which, overall, have a bigger impact on the predicted affinity. The bottom right picture represents the ligand in licorice. The bottom right picture depicts the ligand and the amino acids with the biggest contribution to the affinity represented in licorice.

The molecular dynamics simulations and the MM/PB(GB)SA resulted in four molecules with higher or comparable binding affinities to the reference ligands, 3-hydroxy-C10-HSL and C10-HSL. These molecules were pimozide (Figure 9) from the ZINC/FDA database and CMLDID17434 (Figure 10), CMLDID50121 (Figure 11) and CMLDID60399 (Figure 12) from Chemotheca.

## 4. Conclusions

Because microorganisms embedded in biofilms have several advantages, infections associated with biofilms have been accepted as a significant danger to our society. The recalcitrance of these structures towards existing antimicrobial approaches made necessary the discovery of novel methods to inhibit their mechanisms of formation. Inhibiting the formation of biofilms by disrupting quorum-sensing is the most promising strategy. With that in mind, this work was to use computer-assisted drug design to find promising molecules to block quorum-sensing and, therefore, prevent biofilm formation. This was achieved by optimizing molecular docking, and virtual screening protocols focused on the quorum-sensing receptor from *Chromobacterium violaceum*, CviR.

The optimized molecular docking and virtual screening protocols were applied to two databases, which generated multiple promising molecules that were then analyzed via molecular dynamics simulations and MM/PB(GB)SA calculations, finally resulting in four compounds with higher potential for blocking quorum-sensing and 16 other promising candidates. More experimental studies are required to validate these promising molecules as CviR binders and to confirm them as antagonists.

## Figures and Tables

**Table 1:** Available structures of CviR on PDB.

PDB Code	Protein	Resolution	Ligand	Strain	Reference
3QP1	Ligand-binding domain	1.55 Å	C6-HSL	Strain 31532	[20]
3QP2	Ligand-binding domain	1.638 Å	C8-HSL	Strain 31532	
3QP4	Ligand-binding domain	1.55 Å	C10-HSL	Strain 31532	
3QP5	Full protein	3.249 Å	CL	Strain 31532	
3QP6	Full protein	2 Å	C6-HSL	Strain 12472	
3QP8	Ligand-binding domain	1.6 Å	C10-HSL	Strain 12472	

**Table 2:** Redocking RMSDs for all available structures of CviR and all molecular docking programs used.

Redocking RMSD (Å)								
PDB Code	Ligand	Autodock4	Vina	ASP	CHEMPLP	CHEMSCORE	GOLDSCORE	LeDock
3QP1	C6-HSL	1.08	0.64	0.87	0.70	0.65	0.56	0.49
3QP2a	C8-HSL	1.52	0.84	0.51	0.67	0.73	0.48	0.27
3QP2b	C8-HSL	1.58	0.9	0.69	0.67	0.80	0.74	1.17
3QP4	C10-HSL	1.72	2.04	0.64	1.18	1.05	1.16	1.03
3QP5	CL	1.51	1.38	2.04	1.64	1.58	1.75	1.09
3QP6	C6-HSL	0.96	5.57 *	1.02	0.53	0.59	0.34	0.61
3QP8	C10-HSL	1.74	0.95	0.96	1.03	1.11	0.94	0.51
Average		1.44	1.13	0.96	0.92	0.93	0.85	0.74

* outlier corresponding to ligand binding in an inverted pose.

**Table 3:** Average of the evaluation metrics for active/decoys discrimination ability by program/scoring function.

Active vs. Decoys vs. Programs						
Ev. Metric	Vina	ASP	CHEMPLP	CHEMSCORE	GOLDSCORE	LeDock
AUC	75.2	56.1	69.8	67.2	65.3	70.5
EF 1%	3.1	0.3	4.7	2.2	3.2	0.3
EF 5%	3.3	1.4	4.5	1.9	2.1	2.7
EF 20%	2.5	1.5	2.3	2.0	1.7	2.2
Total gain	0.3	0.1	0.3	0.2	0.2	0.3
BEDROC	0.2	0.1	0.2	0.1	0.1	0.1

**Table 4:** Average of the evaluation metrics for active/decoys discrimination ability, by structure.

Active vs. Decoys vs. Structures							
Ev. Metric	3QP1	3QP2a	3QP2b	3QP4	3QP5	3QP6	3QP8
AUC	67.3	67.0	67.2	69.6	54.9	73.0	72.5
EF 1%	2.6	1.5	2.1	2.6	0.7	3.3	3.3
EF 5%	2.3	2.8	3.4	3.3	0.7	3.0	3.2
EF 20%	1.9	2.0	2.0	2.2	1.1	2.5	2.4
Total gain	0.2	0.2	0.2	0.3	0.1	0.3	0.3
BEDROC	0.1	0.1	0.2	0.2	0.1	0.2	0.2

**Table 5:** Predicted active compounds from the top 25 results in each virtual screening procedure.

Program	Vina		CHEMPLP		LeDock	
Structure	3QP4	3QP6	3QP4	3QP6	3QP4	3QP6
Predicted actives	1	3	2	3	1	1

**Table 6:** Average RMSD values (Å), RMSF (Å), average SASA (Å^2^), percentage of potential ligand SASA buried and an average number of hydrogen bonds for the ligands for the last 40 ns of the simulation of the CviR-ligand complexes.

Database	Ligand	Average RMSD (Å)	RMSF (Å)	Average SASA (Å^2^)	Percentage of Ligand SASA Buried (%)	Average # H-Bonds
Reference	3-Hydroxy-C10-HSL	1.0 ± 0.3	0.7	37 ± 11	92.9	1.9 ± 1.1
	C10-HSL	1.0 ± 0.2	0.7	35 ± 13	93.4	1.5 ± 0.9
ZINC/FDA	Atovaquone	1.2 ± 0.2	0.5	97 ± 21	84.1	0.9 ± 0.7
	Famotidine	2.4 ± 0.2	1.5	90 ± 28	83.1	0.8 ± 0.8
	Glycerol phenylbutyrate	3.2 ± 0.3	1.6	215 ± 23	74.4	0.1 ± 0.1
	Iloprost	2.0 ± 0.3	1.3	203 ± 28	76.1	0.9 ± 0.8
	Mebendazole	0.8 ± 0.2	0.5	101 ± 21	80.6	0.0 ± 0.2
	Mirabegron	2.2 ± 0.2	1.5	91 ± 23	86.9	0.4 ± 0.6
	Montelukast	1.9 ± 0.5	1.3	245 ± 29	73.9	0.5 ± 0.7
	Paliperidone	1.1 ± 0.3	1.0	208 ± 26	69.1	0.2 ± 0.4
	Pimozide	2.4 ± 0.2	1.0	42 ± 12	93.8	1.1 ± 0.6
	Sulfasalazine	1.7 ± 0.5	1.0	110 ± 18	82.4	0.9 ± 0.8
Mu.Ta.Lig Chemotheca	CMLDID17434	2.6 ± 0.2	1.2	177 ± 26	77.0	1.2 ± 0.7
	CMLDID18049	2.1 ± 0.1	1.2	178 ± 28	73.5	0.3 ± 0.5
	CMLDID23812	2.5 ± 0.4	1.7	168 ± 25	77.9	1.1 ± 0.7
	CMLDID2574	1.1 ± 0.3	1.1	168 ± 31	71.7	0.1 ± 0.3
	CMLDID35542	2.1 ± 0.2	1.3	67 ± 21	89.5	0.7 ± 0.8
	CMLDID38590	3.2 ± 0.3	1.9	192 ± 28	75.6	1.4 ± 0.9
	CMLDID40723	1.6 ± 0.4	1.1	118 ± 35	80.1	0.0 ± 0.2
	CMLDID50121	2.4 ± 0.4	1.6	129 ± 24	81.2	0.8 ± 0.8
	CMLDID5450	2.9 ± 0.4	2.0	188 ± 42	76.2	1.2 ± 0.8
	CMLDID60399	1.3 ± 0.2	0.7	88 ± 12	86.9	1.0 ± 0.9

**Table 7:** Results for the MM/PB(GB)SA calculations and all components.

		MM		GBSA		PBSA			∆G_binding_	
Database	Ligand	E_vdw_ (kcal/mol)	E_el_ (kcal/mol)	E_SURF_ (kcal/mol)	E_GB_ (kcal/mol)	E_npolar_ (kcal/mol)	E_disper_ (kcal/mol)	E_PB_ (kcal/mol)	MM/GBSA (kcal/mol)	MM/PBSA (kcal/mol)
Reference	3-Hydroxy-C10-HSL	−42.9 ± 0.2	−49.0 ± 0.4	−6.1 ± 0.0	46.8 ± 0.2	−31.9 ± 0.0	51.6 ± 0.0	12.6 ± 0.0	−51.1 ± 0.2	−59.6 ± 0.2
	C10-HSL	−43.4 ± 0.1	−37.0 ± 0.2	−6.1 ± 0.0	37.4 ± 0.1	−31.9 ± 0.0	50.5 ± 0.0	10.1 ± 0.0	−49.0 ± 0.2	−51.7 ± 0.2
ZINC/FDA	Atovaquone	−40.0 ± 0.2	−28.0 ± 0.2	−5.7 ± 0.0	34.8 ± 0.1	−31.8 ± 0.1	54.7 ± 0.1	9.8 ± 0.0	−38.9 ± 0.2	−35.3 ± 0.2
	Famotidine	−37.8 ± 0.2	−33.9 ± 0.4	−5.7 ± 0.0	47.9 ± 0.3	−26.2 ± 0.1	46.3 ± 0.1	12.8 ± 0.1	−29.6 ± 0.2	−38.8 ± 0.2
	Glycerol phenylbutyrate	−60.4 ± 0.1	−9.2 ± 0.1	−8.1 ± 0.0	34.4 ± 0.1	−43.4 ± 0.1	72.5 ± 0.1	9.2 ± 0.0	−43.3 ± 0.1	−31.3 ± 0.2
	Iloprost	−57.1 ± 0.2	−26.3 ± 0.3	−8.1 ± 0.0	43.2 ± 0.2	−43.6 ± 0.1	72.2 ± 0.1	11.6 ± 0.1	−48.4 ± 0.2	−43.2 ± 0.2
	Mebendazole	−34.6 ± 0.1	−4.5 ± 0.2	−4.8 ± 0.0	21.2 ± 0.2	−25.3 ± 0.1	44.3 ± 0.1	5.0 ± 0.1	−22.7 ± 0.1	−15.1 ± 0.1
	Mirabegron	−50.2 ± 0.2	−20.7 ± 0.4	−6.9 ± 0.0	40.1 ± 0.3	−37.4 ± 0.1	62.4 ± 0.1	10.5 ± 0.1	−37.7 ± 0.2	−35.4 ± 0.2
	Montelukast	−57.8 ± 0.2	−26.5 ± 1.6	−7.2 ± 0.0	48.5 ± 1.5	−42.1 ± 0.1	73.6 ± 0.1	13.1 ± 0.4	−43.0 ± 0.3	−39.7 ± 0.2
	Paliperidone	−42.4 ± 0.2	−14.5 ± 0.3	−5.3 ± 0.0	31.2 ± 0.2	−30.3 ± 0.1	53.7 ± 0.1	7.8 ± 0.1	−31.0 ± 0.2	−25.7 ± 0.2
	Pimozide	−55.9 ± 0.2	−86.5 ± 0.4	−7.5 ± 0.0	82.6 ± 0.4	−42.7 ± 0.1	73.6 ± 0.1	21.9 ± 0.1	−67.2 ± 0.2	−89.6 ± 0.2
	Sulfasalazine	−47.5 ± 0.2	−33.9 ± 0.3	−6.5 ± 0.0	47.9 ± 0.2	−32.6 ± 0.1	57.2 ± 0.1	12.0 ± 0.1	−40.1 ± 0.2	−44.7 ± 0.2
Mu.Ta.Lig Chemotheca	CMLDID17434	−53.6 ± 0.2	−58.1 ± 0.5	−7.1 ± 0.0	68.8 ± 0.5	−39.3 ± 0.1	65.8 ± 0.1	17.1 ± 0.1	−50.0 ± 0.2	−68.1 ± 0.2
	CMLDID18049	−42.0 ± 0.2	−8.2 ± 0.4	−5.5 ± 0.0	27.9 ± 0.3	−30.1 ± 0.1	53.9 ± 0.1	6.5 ± 0.1	−27.8 ± 0.2	−20.0 ± 0.2
	CMLDID23812	−53.6 ± 0.2	−29.0 ± 0.2	−7.4 ± 0.0	42.0 ± 0.2	−39.7 ± 0.1	65.8 ± 0.1	10.7 ± 0.1	−48.0 ± 0.2	−45.8 ± 0.2
	CMLDID2574	−37.2 ± 0.2	−0.9 ± 0.2	−4.8 ± 0.0	17.6 ± 0.1	−26.6 ± 0.1	47.5 ± 0.1	4.2 ± 0.0	−25.4 ± 0.1	−13.0 ± 0.1
	CMLDID35542	−53.2 ± 0.2	−35.4 ± 0.3	−6.9 ± 0.0	51.0 ± 0.2	−35.6 ± 0.1	62.2 ± 0.1	13.1 ± 0.1	−44.4 ± 0.2	−48.8 ± 0.2
	CMLDID38590	−52.5 ± 0.2	−15.8 ± 0.1	−7.2 ± 0.0	28.2 ± 0.1	−39.4 ± 0.1	65.6 ± 0.1	6.9 ± 0.2	−47.3 ± 0.2	−32.3 ± 0.2
	CMLDID40723	−45.0 ± 0.2	−3.4 ± 0.2	−5.5 ± 0.0	19.8 ± 0.2	−30.4 ± 0.1	53.0 ± 0.2	5.8 ± 0.1	−34.1 ± 0.2	−20.1 ± 0.2
	CMLDID50121	−51.6 ± 0.2	−62.4 ± 0.1	−6.6 ± 0.0	75.2 ± 0.5	−34.0 ± 0.1	60.5 ± 0.1	20.1 ± 0.1	−45.4 ± 0.2	−67.4 ± 0.2
	CMLDID5450	−53.9 ± 0.2	−34.7 ± 0.3	−7.4 ± 0.0	47.7 ± 0.3	−39.8 ± 0.1	67.7 ± 0.2	11.8 ± 0.1	−48.4 ± 0.2	−48.9 ± 0.2
	CMLDID60399	−56.7 ± 0.2	−45.4 ± 0.3	−7.1 ± 0.0	56.1 ± 0.2	−36.8 ± 0.4	62.8 ± 0.1	14.2 ± 0.0	−53.0 ± 0.2	−61.8 ± 0.2

**Table 8:** Average RMSD values (Å), RMSF (Å), average SASA (Å2), percentage of potential ligand SASA buried and average number of hydrogen bonds for the ligands for the last 40 ns of the simulation of the replicas.

Database	Ligand	Replica	Average RMSD (Å)	RMSF (Å)	Average SASA (Å^2^)	Percentage of Ligand SASA Buried (%)	Average # H-Bonds
ZINC/FDA	Pimozide	Original	2.4 ± 0.2	1.0	42 ± 12	93.8	1.1 ± 0.6
		1	1.9 ± 0.5	1.0	70 ± 42	90.0	1.0 ± 0.6
		2	2.1 ± 0.1	0.9	32 ± 9	95.2	1.1 ± 0.6
		3	1.5 ± 0.6	1.1	29 ± 33	95.9	1.2 ± 0.6
Mu.Ta.Lig Chemotheca	CMLDID17434	Original	2.6 ± 0.2	1.2	177 ± 26	77.0	1.2 ± 0.7
		1	2.9 ± 0.4	1.4	170 ± 20	78.4	1.1 ± 0.8
		2	3.1 ± 0.4	1.3	181 ± 24	76.7	1.3 ± 0.7
		3	2.4 ± 0.5	1.5	179 ± 24	77.1	1.3 ± 0.7
	CMLDID50121	Original	2.4 ± 0.4	1.6	129 ± 24	81.2	0.8 ± 0.8
		1	2.4 ± 0.3	1.1	150 ± 28	78.5	0.8 ± 0.7
		2	2.1 ± 0.7	1.4	142 ± 22	79.5	0.8 ± 0.7
		3	2.2 ± 0.6	1.4	152 ± 25	78.1	0.7 ± 0.7
	CMLDID60399	Original	1.3 ± 0.2	0.7	88 ± 12	86.9	1.0 ± 0.9
		1	0.9 ± 0.2	0.5	90 ± 15	86.6	1.0 ± 0.8
		2	1.0 ± 0.2	0.6	93 ± 13	86.2	1.0 ± 0.9
		3	1.7 ± 0.4	1.0	87 ± 23	87.2	1.1 ± 1.0

## Supplementary Materials

The following are available online, Figure S1. Known actives for CviR, Figure S2. Comparison of the Vina scores of the 10 chosen molecules from the ZINC/FDA library and the known actives, Figure S3. Comparison of the GOLD/CHEMPLP scores of the 10 chosen molecules from the ZINC/FDA library and the known actives, Figure S4. Comparison of the LeDock scores of the 10 chosen molecules from the ZINC/FDA library and the known actives, Figure S5. Comparison of the Vina scores of the 10 chosen molecules from the Chemotheca and the known actives, Figure S6. Comparison of the GOLD/CHEMPLP scores of the 10 chosen molecules from the Chemotheca and the known actives, Figure S7. Comparing the LeDock scores of the 10 chosen molecules from the Chemotheca and the known actives, Figure S8. Root-mean-square deviation plots of the Cα atoms for the CviR-ligand complexes for the selected molecules from the ZINC/FDA database, Figure S9. Root mean square deviation plots of the ligands on the CviR-ligand complexes for the selected molecules from the ZINC/FDA database, Figure S10. Root mean square deviation plots of the Cα atoms for the CviR-ligand complexes for the selected molecules from the Chemotheca database, Figure S11. Root-mean-square deviation plots of the ligands on the CviR-ligand complexes for the selected molecules from the Chemotheca database, Table S1. Redocking scores for all available structures of CviR and all molecular docking programs used, Table S2. Cross-docking Results for every structure with all programs used, Table S3. RMSD for the cross-docking procedure, Table S4. Average RMSD values (Å) of the last 40ns of the simulation for the CviR-ligand complexes for the selected molecules.
