# Supplementary material for: Identification of New Potential Inhibitors of Quorum Sensing through a Specialized Multi-Level Computational Approach
Source: Molecules. 2021 Apr 29;26(9):2600. doi: 10.3390/molecules26092600 (PMC8125606; doi:10.3390/molecules26092600)
Supplement: Supplementary file 1 [file molecules-26-02600-s001.zip › molecules-1160327-supplementary.pdf]

Supplementary Materials

Identification of New Quorum Sensing Inhibitors through a Specialized Multi-Level Computational Approach

Fábio G. Martins [1,2], André Melo [2] and Sérgio F. Sousa [1,*]

[1] UCIBIO/REQUIMTE, BioSIM—Departamento de Biomedicina, Faculdade de Medicina da Universidade do Porto, 4200-319 Porto, Portugal; fabmaru@gmail.com
[2] LAQV/REQUIMTE, Departamento de Química e Bioquímica, Faculdade de Ciências da Universidade do Porto, 4169-007 Porto, Portugal; asmelo@fc.up.pt
* Correspondence: sergiofsousa@med.up.pt

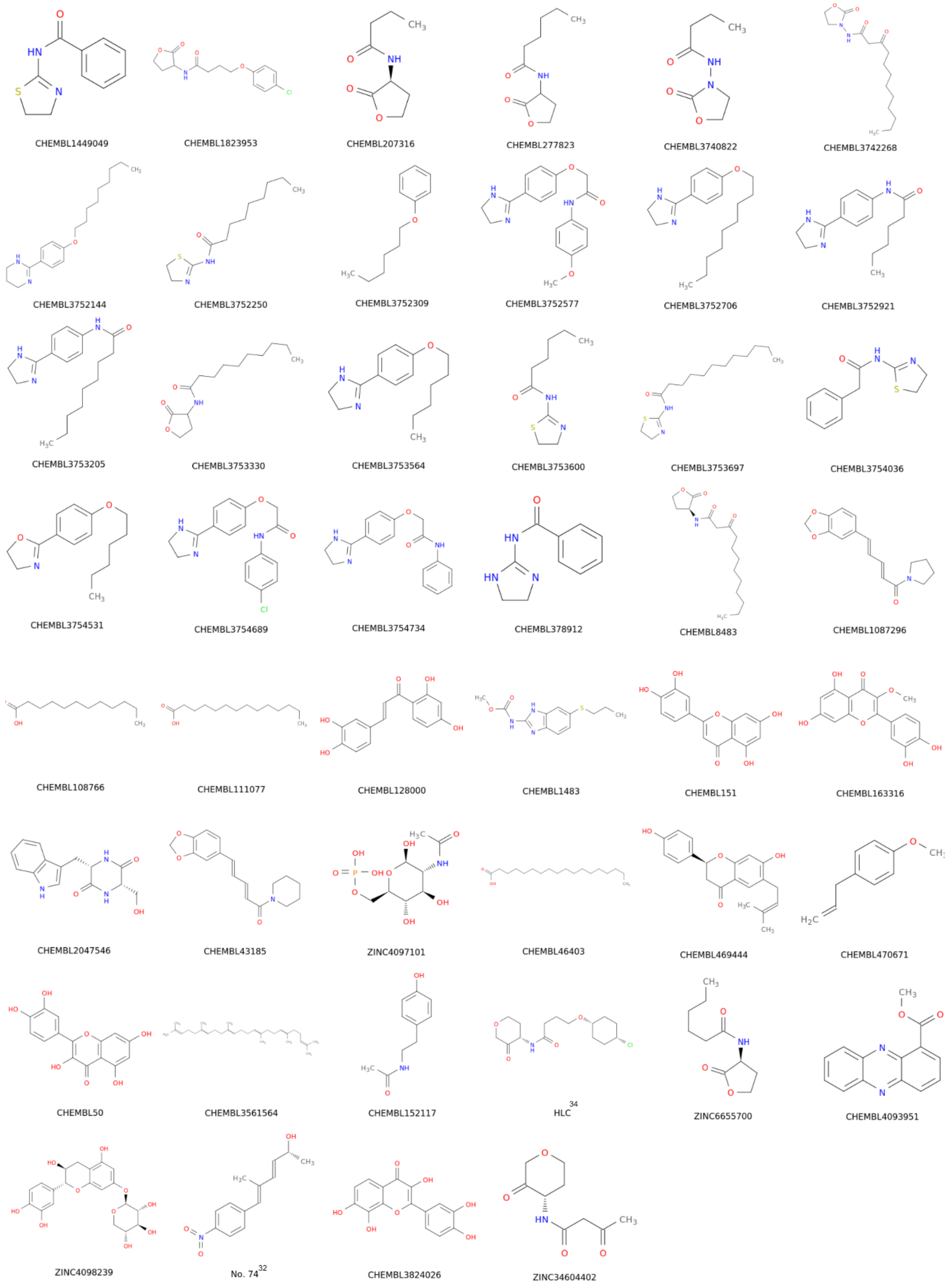

Figure S1 - Known actives for CviR

Table S1 - Redocking scores for all available structures of CviR and all molecular docking programs used.

Redocking Score							
PDB Code	Autodock 4 (kcal/mol)	Vina (kcal/mol)	ASP	CHEMPLP	CHEMSCORE	GOLDSCORE	LeDock (kcal/mol)
3QP1	-7.9	-7.7	38.1	70.3	32.8	57.3	-6
3QP2a	-8.4	-7.9	40.7	77.6	35.9	62.5	-6.4
3QP2b	-8.4	-7.9	42.1	76.6	36	64	-6.4
3QP4	-9.1	-8.2	43.9	87.2	38.8	68.9	-6.9
3QP5	-8.4	-8.1	43.9	72.6	33.6	62.2	-6.3
3QP6	-7.9	-7.8	39.3	71.7	33.6	57.8	-5.9
3QP8	-9.1	-8.2	45.9	87.3	39.5	72.5	-6.8

Table S2 - Crossdocking Results for every structure with all programs used.

			Crossdocking Results						
Program	PDB Code	Ligand	3QP1	3QP2a	3QP2b	3QP4	3QP5	3QP6	3QP8
Autodock 4	3QP1	C6-HSL	-7.9	-7.8	-8.8	-7.9	-6.8	-7.9	-7.8
	3QP2	C8-HSL	-8.4	-8.4	-8.5	-8.5	-7.5	-8.8	-8.6
	3QP4	C10-HSL	-9.0	-8.9	-9.1	-9.1	-8.0	-9.2	-8.9
	3QP5	CL	-9.1	-9.5	-9.4	-9.4	-8.4	-9.9	-9.7
	3QP6	C6-HSL	-7.9	-7.8	-7.8	-7.9	-7.2	-7.9	-7.9
	3QP8	C10-HSL	-8.9	-8.9	-9.0	-9.0	-7.9	-9.3	-9.0
Vina	3QP1	C6-HSL	-7.7	-7.6	-7.6	-7.8	-6.7	-7.7	-7.6
	3QP2	C8-HSL	-8.0	-7.9	-7.9	-8.0	-7.3	-8.0	-7.9
	3QP4	C10-HSL	-8.2	-8.2	-8.2	-8.2	-7.5	-8.3	-8.1
	3QP5	CL	-8.4	-8.9	-8.9	-8.9	-8.1	-8.8	-9.0
	3QP6	C6-HSL	-7.8	-7.7	-7.6	-7.7	-6.7	-7.7	-7.6
	3QP8	C10-HSL	-8.2	-8.3	-8.2	-8.2	-7.4	-8.3	-8.2
ASP	3QP1	C6-HSL	38.2	37.8	37.1	37.5	35.1	38.9	39.5
	3QP2	C8-HSL	41.7	40.9	41.9	41.7	37.7	43.8	43.2
	3QP4	C10-HSL	44.5	43.9	43.7	44.8	40.5	46.3	45.6
	3QP5	CL	48.2	49.3	48.3	48.6	44.3	49.5	51.0
	3QP6	C6-HSL	37.6	37.1	37.6	37.6	33.7	40.2	39.6
	3QP8	C10-HSL	43.8	43.4	44.0	44.4	39.6	46.5	45.5
CHEMPLP	3QP1	C6-HSL	69.3	69.2	68.9	70.3	58.8	71.2	70.5
	3QP2	C8-HSL	77.5	77.2	77.9	76.7	65.0	80.6	78.4
	3QP4	C10-HSL	83.9	84.1	85.1	85.5	73.5	88.7	85.6
	3QP5	CL	83.7	85.5	86.0	84.5	70.9	80.5	88.2
	3QP6	C6-HSL	69.0	69.3	69.4	70.4	58.7	71.2	70.1
	3QP8	C10-HSL	82.6	84.0	85.5	86.2	71.5	88.9	86.2
GOLDSCORE	3QP1	C6-HSL	57.7	56.9	56.6	58.7	50.1	58.7	59.1
	3QP2	C8-HSL	65.3	61.9	62.1	63.9	51.8	64.2	64.6
	3QP4	C10-HSL	63.8	66.9	69.0	70.8	59.1	74.1	73.0
	3QP5	CL	67.0	68.8	69.9	68.5	62.3	74.0	72.2
	3QP6	C6-HSL	56.3	55.8	56.3	56.6	51.3	57.7	57.8
	3QP8	C10-HSL	64.0	66.8	68.3	71.0	58.3	72.3	73.7
CHEMSCORE	3QP1	C6-HSL	32.9	32.3	32.7	32.5	27.4	33.7	33.4
	3QP2	C8-HSL	36.5	35.9	35.9	35.9	29.5	37.0	36.6
	3QP4	C10-HSL	39.7	38.8	38.6	39.3	33.1	39.9	39.4
	3QP5	CL	41.2	41.2	41.1	41.0	33.1	42.3	41.1
	3QP6	C6-HSL	33.1	32.7	32.3	32.4	27.0	33.6	33.5
	3QP8	C10-HSL	39.2	38.7	38.7	39.0	33.9	40.3	39.4
LeDock	3QP1	C6-HSL	-6.0	-5.9	-5.9	-6.0	-5.0	-5.8	-5.8
	3QP2	C8-HSL	-6.5	-6.4	-6.4	-6.5	-5.5	-6.3	-6.3
	3QP4	C10-HSL	-6.4	-6.8	-6.7	-7.0	-5.9	-6.8	-6.7
	3QP5	CL	-6.4	-7.3	-7.2	-7.4	-6.3	-7.3	-7.5
	3QP6	C6-HSL	-6.0	-5.9	-5.9	-6.1	-5.1	-5.9	-5.8
	3QP8	C10-HSL	-6.8	-6.8	-6.7	-7.0	-5.8	-6.9	-6.8

Table S3 - RMSDs for the crossdocking procedure

			Crossdocking RMSD (Å)						
Program	PDB Code	Ligand	3QP1	3QP2a	3QP2b	3QP4	3QP5	3QP6	3QP8
Autodock 4	3QP1	C6-HSL	0.97	1.03	0.97	1.05	1.30	0.92	1.00
	3QP2	C8-HSL	1.18	1.52	1.68	1.16	1.95	1.48	1.54
	3QP4	C10-HSL	1.96	1.77	1.72	1.36	2.29	1.82	2.08
	3QP5	CL	1.78	2.04	1.57	1.64	1.75	1.75	1.92
	3QP6	C6-HSL	0.66	0.68	0.66	0.71	1.08	0.86	0.64
	3QP8	C10-HSL	2.25	1.58	1.49	2.02	1.78	1.56	1.99
Vina	3QP1	C6-HSL	0.63	0.66	0.67	0.55	5.30	5.66	5.84
	3QP2	C8-HSL	1.26	0.92	0.90	0.85	1.52	1.19	1.16
	3QP4	C10-HSL	1.90	1.98	2.05	2.02	2.43	1.43	1.45
	3QP5	CL	1.36	1.31	0.77	0.75	1.36	1.60	1.52
	3QP6	C6-HSL	0.65	0.55	0.41	0.38	5.11	5.57	5.53
	3QP8	C10-HSL	1.95	2.05	2.13	2.00	2.26	1.12	1.09
ASP	3QP1	C6-HSL	0.72	0.75	0.65	0.88	1.75	1.19	0.66
	3QP2	C8-HSL	0.83	0.56	0.73	0.88	1.80	0.70	0.76
	3QP4	C10-HSL	1.86	0.80	1.18	0.63	2.46	0.90	1.10
	3QP5	CL	1.28	1.21	1.50	1.22	2.45	1.26	1.30
	3QP6	C6-HSL	1.14	1.17	0.72	1.14	1.17	0.73	0.70
	3QP8	C10-HSL	1.28	1.00	1.16	0.77	1.44	0.92	1.06
CHEMPLP	3QP1	C6-HSL	0.62	0.76	0.76	0.79	0.80	0.47	0.91
	3QP2	C8-HSL	0.60	0.76	0.65	0.44	1.83	0.50	0.45
	3QP4	C10-HSL	1.45	1.60	1.32	1.32	2.58	0.78	1.32
	3QP5	CL	1.13	1.20	1.07	0.92	1.53	1.36	1.19
	3QP6	C6-HSL	0.89	0.86	0.78	0.71	1.03	0.78	0.75
	3QP8	C10-HSL	1.26	1.30	1.32	1.09	1.28	0.86	0.86
GOLDSCORE	3QP1	C6-HSL	0.62	0.72	0.61	0.60	1.82	0.65	0.62
	3QP2	C8-HSL	0.71	0.52	0.72	0.81	0.63	0.71	0.77
	3QP4	C10-HSL	1.37	0.96	1.32	1.22	1.27	1.00	1.25
	3QP5	CL	0.84	1.72	1.57	1.21	1.76	1.22	1.21
	3QP6	C6-HSL	0.70	0.41	0.52	0.87	1.53	0.53	0.29
	3QP8	C10-HSL	0.96	1.19	0.98	1.17	0.98	0.89	0.79
CHEMSCORE	3QP1	C6-HSL	0.64	0.65	1.18	0.70	0.96	0.53	0.63
	3QP2	C8-HSL	0.69	0.76	0.69	0.73	1.07	0.79	0.74
	3QP4	C10-HSL	1.31	0.83	1.18	1.22	2.44	1.38	1.27
	3QP5	CL	1.15	1.45	1.08	1.09	1.51	1.29	1.12
	3QP6	C6-HSL	0.85	0.89	0.84	0.77	1.05	0.80	0.56
	3QP8	C10-HSL	1.46	0.71	1.17	0.95	2.45	0.77	0.99
LeDock	3QP1	C6-HSL	0.58	0.74	0.66	0.73	0.69	0.83	0.58
	3QP2	C8-HSL	1.10	0.29	1.08	0.50	0.74	0.71	0.54
	3QP4	C10-HSL	1.11	1.08	1.04	0.97	1.31	1.01	0.79
	3QP5	CL	1.19	1.44	0.63	0.80	1.69	1.07	1.41
	3QP6	C6-HSL	0.56	0.55	0.49	0.53	0.54	0.69	0.33
	3QP8	C10-HSL	1.15	0.61	0.71	0.98	1.21	0.74	0.79

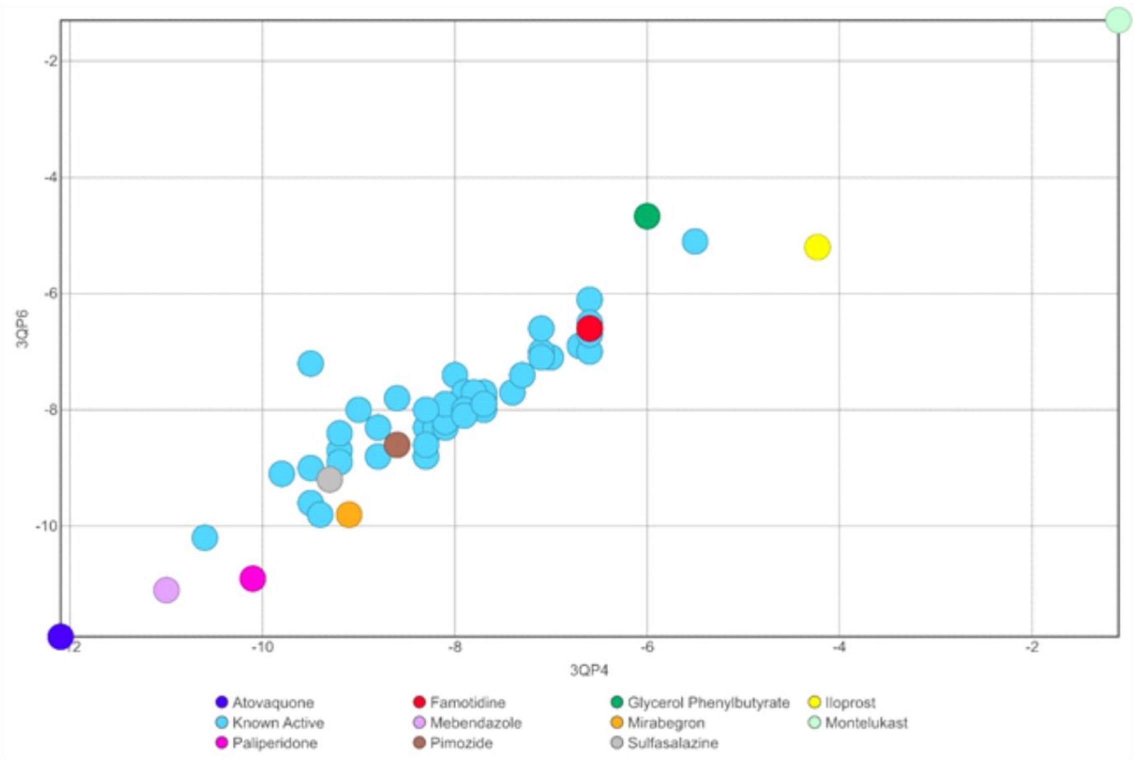

Figure S2 - Comparison of the Vina scores of the 10 chosen molecules from the ZINC/FDA library and the known actives.

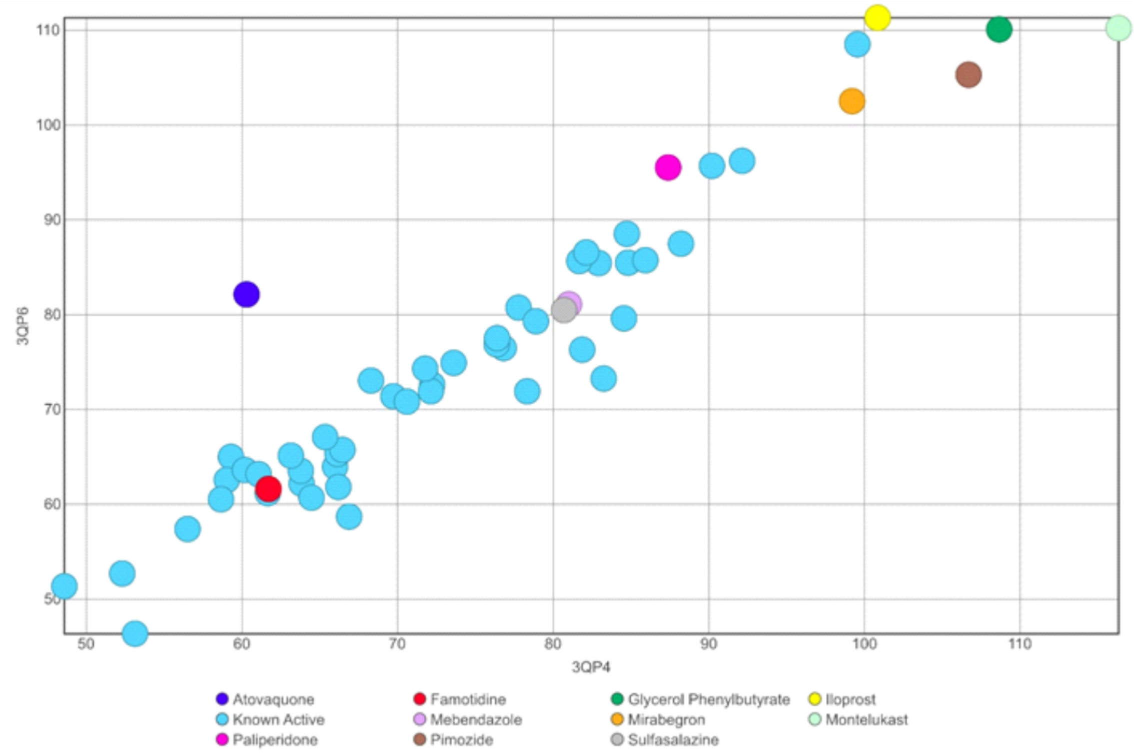

Figure S3 - Comparison of the GOLD/CHEMPLP scores of the 10 chosen molecules from the ZINC/FDA library and the known actives.

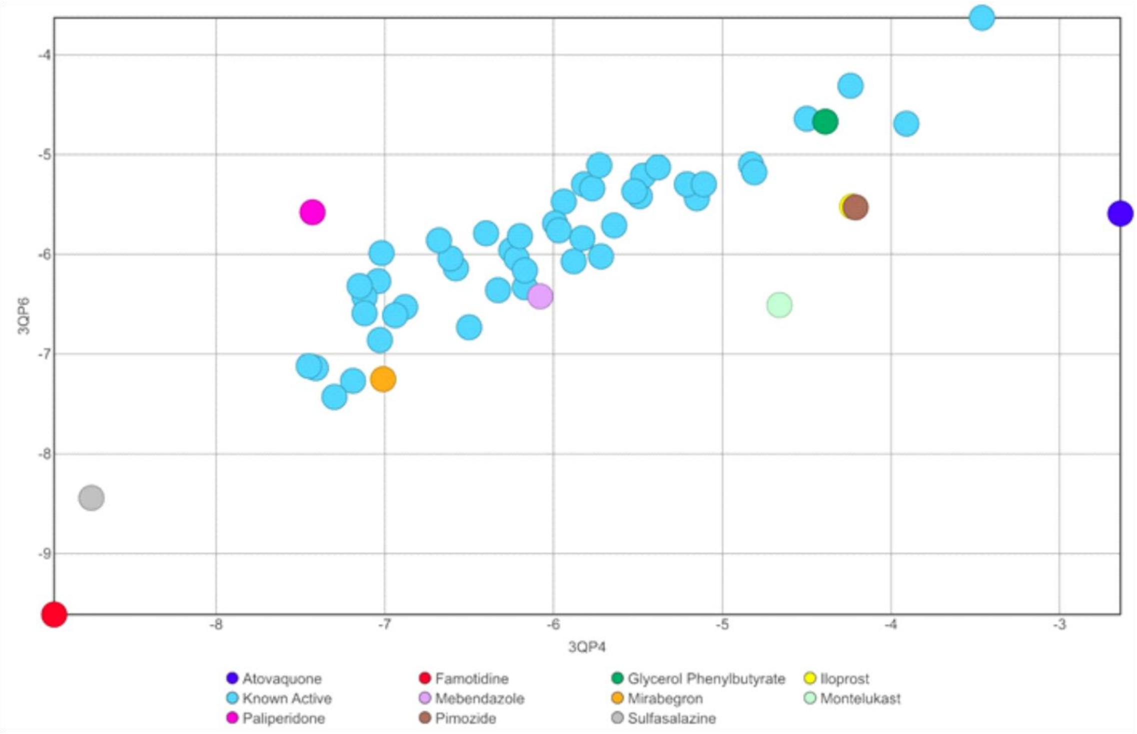

Figure S4 - Comparison of the LeDock scores of the 10 chosen molecules from the ZINC/FDA library and the known actives.

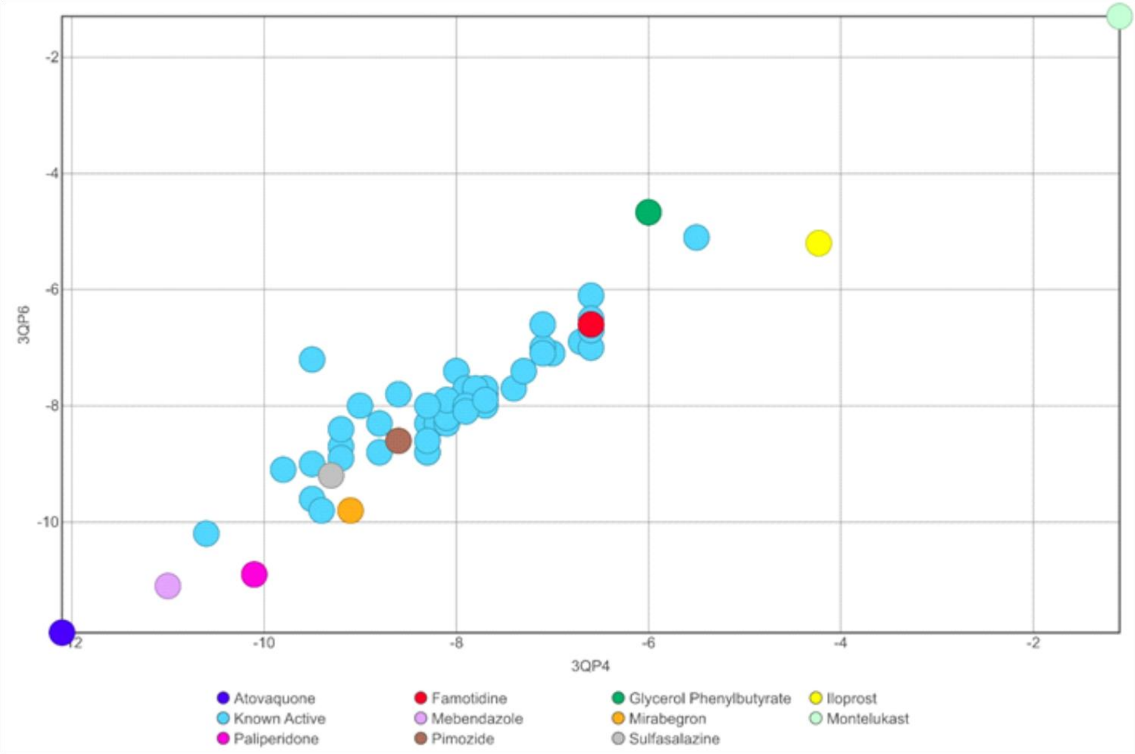

Figure S5 - Comparison of the Vina scores of the 10 chosen molecules from the Chemotheca and the known actives.

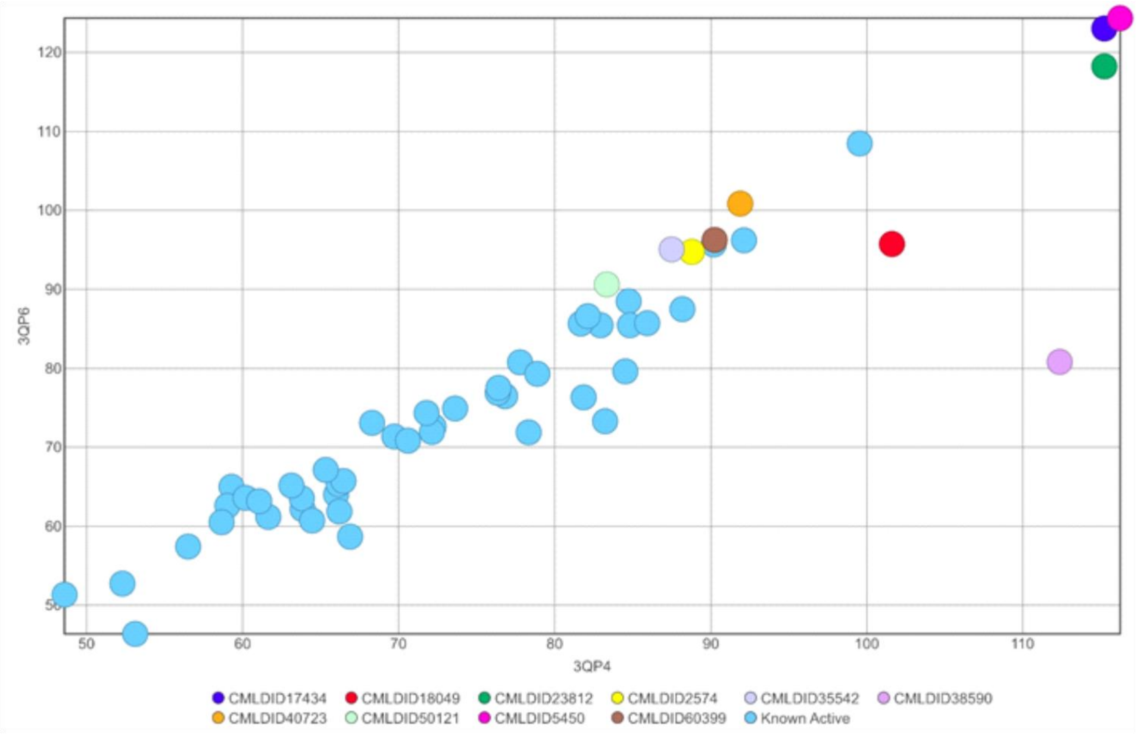

Figure S6 - Comparison of the GOLD/CHEMPLP scores of the 10 chosen molecules from the Chemotheca and the known actives.

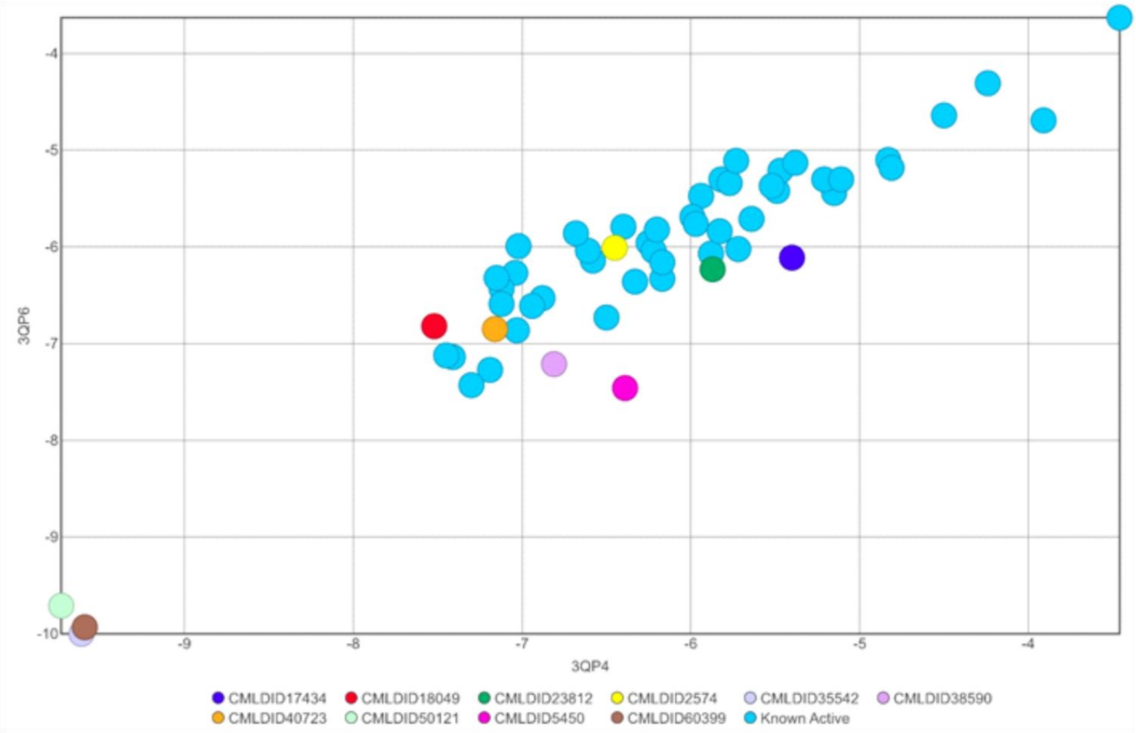

Figure S7 - Comparison of the LeDock scores of the 10 chosen molecules from the Chemotheca and the known actives.

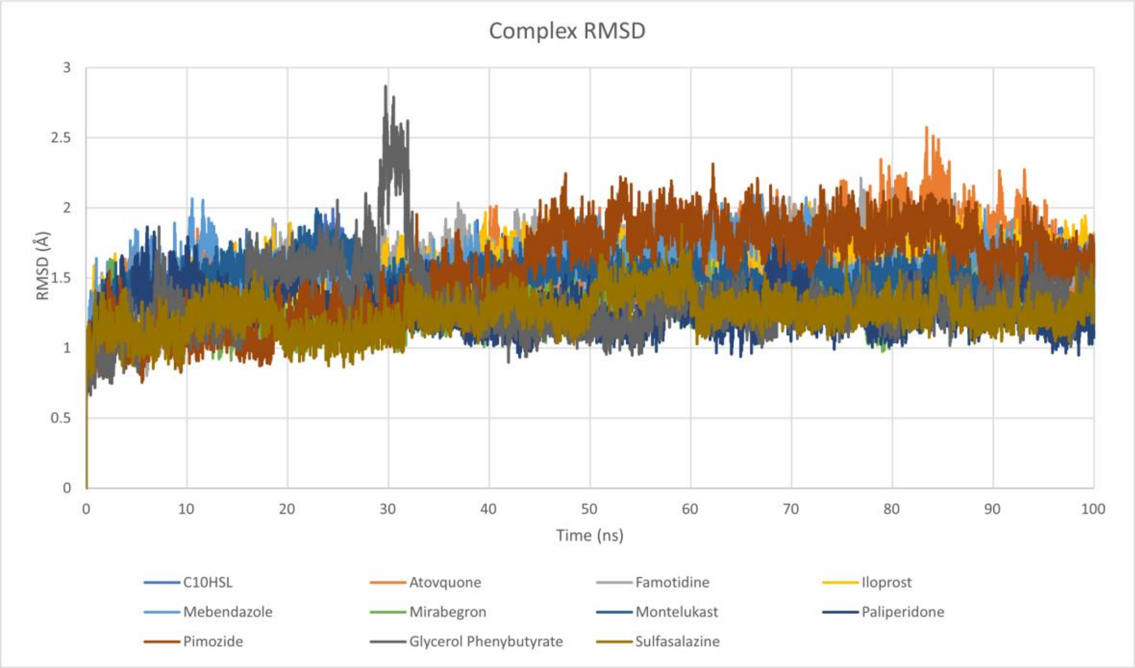

Figure S8 - Root mean square deviation plots of the Cα atoms for the CviR-ligand complexes for the selected molecules from the ZINC/FDA database.

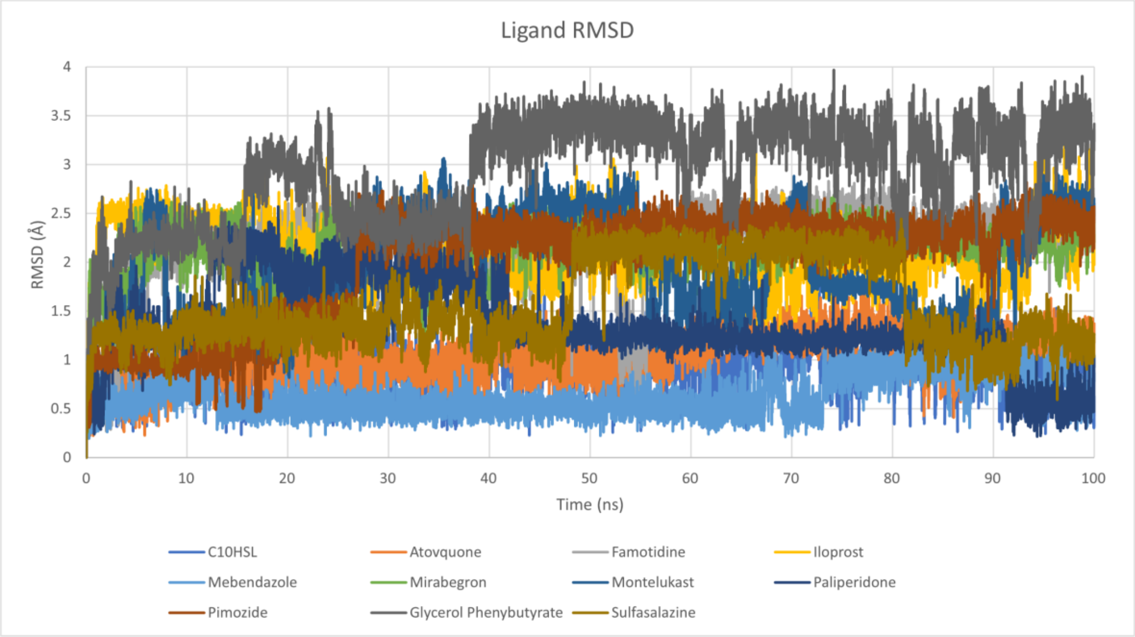

Figure S9 - Root mean square deviation plots of the ligands on the CviR-ligand complexes for the selected molecules from the ZINC/FDA database.

Table S4 - Average RMSD values (Å) of the last 40ns of the simulation for the CviR-ligand complexes for the selected molecules.

Database	Ligand	Average RMSD (Å)
Reference	C10HSL	1.55 ± 0.12
ZINC/FDA	Atovaquone	1.78 ± 0.19
	Famotidine	1.68 ± 0.14
	Iloprost	1.72 ± 0.08
	Mebendazole	1.64 ± 0.14
	Mirabegron	1.35 ± 0.09
	Montelukast	1.50 ± 0.09
	Paliperidone	1.26 ± 0.13
	Pimozide	1.78 ± 0.15
	Glycerol Phenylbutyrate	1.29 ± 0.10
	Sulfasalazine	1.27 ± 0.09
Mu.Ta.Lig Chemotheca	CMLDID17434	1.50 ± 0.12
	CMLDID18049	1.31 ± 0.13
	CMLDID23812	1.39 ± 0.16
	CMLDID2574	1.32 ± 0.14
	CMLDID35542	1.34 ± 0.10
	CMLDID38590	1.41 ± 0.14
	CMLDID40723	1.42 ± 0.13
	CMLDID50121	1.37 ± 0.16
	CMLDID5450	1.46 ± 0.09
	CMLDID60399	1.29 ± 0.07

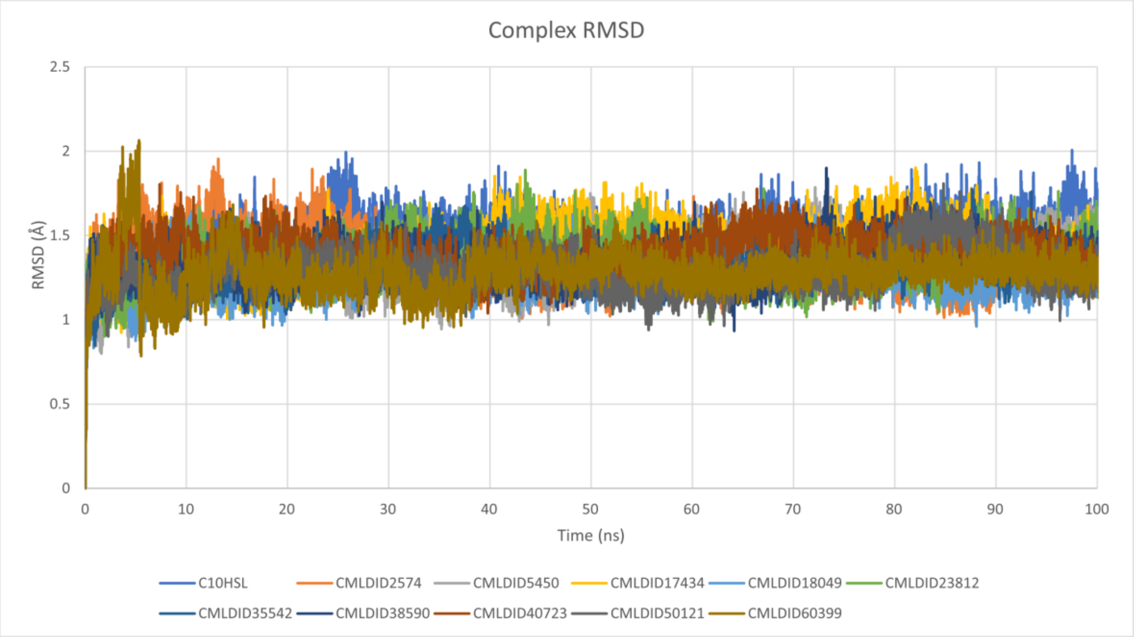

Figure S10 - Root mean square deviation plots of the Cα atoms for the CviR-ligand complexes for the selected molecules from the Chemotheca database.

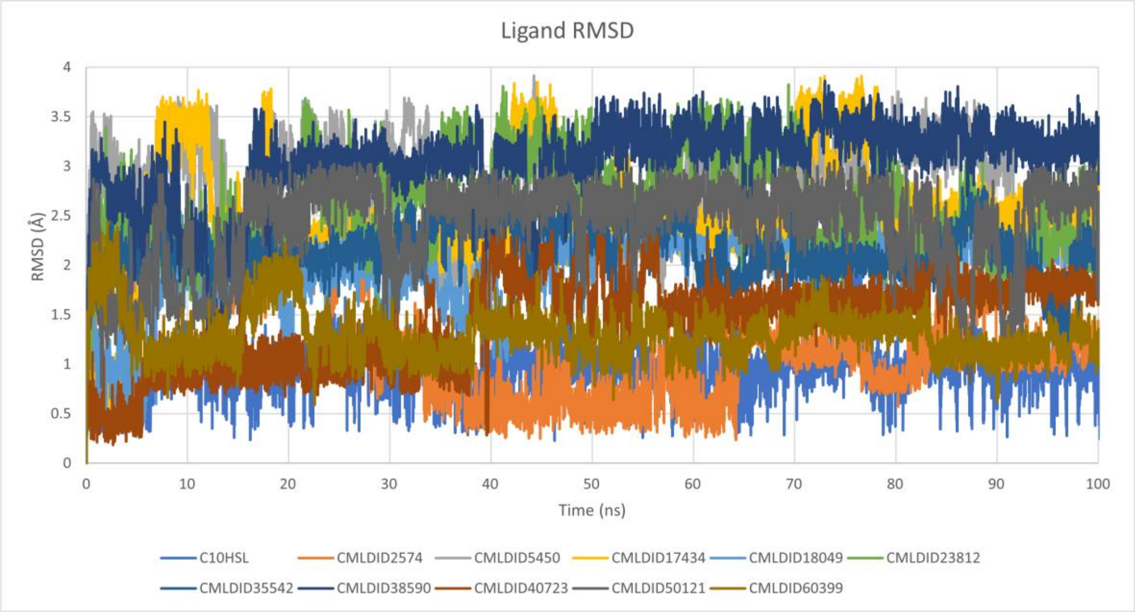

Figure S11 - Root mean square deviation plots of the ligands on the CviR-ligand complexes for the selected molecules from the Chemotheca database
